# The cryo-EM structure of the *bd* oxidase from *M. tuberculosis* reveals a unique structural framework and enables rational drug design to combat TB

**DOI:** 10.1038/s41467-021-25537-z

**Published:** 2021-09-02

**Authors:** Schara Safarian, Helen K. Opel-Reading, Di Wu, Ahmad R. Mehdipour, Kiel Hards, Liam K. Harold, Melanie Radloff, Ian Stewart, Sonja Welsch, Gerhard Hummer, Gregory M. Cook, Kurt L. Krause, Hartmut Michel

**Affiliations:** 1 Department of Molecular Membrane Biology, Max Planck Institute of Biophysics, Frankfurt/Main, Germany; 2 Department of Biochemistry, University of Otago, Dunedin, New Zealand; 3 Department of Theoretical Biophysics, Max Planck Institute of Biophysics, Frankfurt/Main, Germany; 4 Department of Microbiology and Immunology, School of Biomedical Sciences, University of Otago, Dunedin, New Zealand; 5 Department of Chemistry, University of Otago, Dunedin, New Zealand; 6 Central Electron Microscopy Facility, Max Planck Institute of Biophysics, Frankfurt am Main, Germany; 7 Institute of Biophysics, Goethe University Frankfurt, Frankfurt/Main, Germany

**Keywords:** Structural biology, Bioenergetics, Bacterial structural biology, Cryoelectron microscopy

## Abstract

New drugs are urgently needed to combat the global TB epidemic. Targeting simultaneously multiple respiratory enzyme complexes of *Mycobacterium tuberculosis* is regarded as one of the most effective treatment options to shorten drug administration regimes, and reduce the opportunity for the emergence of drug resistance. During infection and proliferation, the cytochrome *bd* oxidase plays a crucial role for mycobacterial pathophysiology by maintaining aerobic respiration at limited oxygen concentrations. Here, we present the cryo-EM structure of the cytochrome *bd* oxidase from *M. tuberculosis* at 2.5 Å. In conjunction with atomistic molecular dynamics (MD) simulation studies we discovered a previously unknown MK-9-binding site, as well as a unique disulfide bond within the Q-loop domain that defines an inactive conformation of the canonical quinol oxidation site in Actinobacteria. Our detailed insights into the long-sought atomic framework of the cytochrome *bd* oxidase from *M. tuberculosis* will form the basis for the design of highly specific drugs to act on this enzyme.

## Abstract (web summary)

*M. tuberculosis* cytochrome *bd* oxidase is of interest as a TB drug target. Here, the authors present the 2.5 Å cryo-EM structure of *M. tuberculosis* cytochrome *bd* oxidase and identify a disulfide bond within the canonical quinol binding and oxidation domain (Q-loop) and a menaquinone-9 binding site at heme *b*_595_.

## Introduction

Tuberculosis (TB) constitutes a major public health problem and is a leading cause of global mortality from infectious diseases^1^. Even in 2020 more people died from TB than from COVID-19. The emergence of extensively drug-resistant (XDR) and totally drug-resistant strains threatens to return us to the pre-antibiotic era for this disease. This fact combined with the very long time required to treat TB has created an urgent need to develop novel and fast-acting drugs that are not vulnerable to existing drug resistance mechanisms. The ATP synthase inhibitor, Bedaquiline (Sirturo), which was approved by the FDA in 2012, was the first drug in 40 years to attack TB by a novel mechanism of action^2–4^. This marked the starting point for exploration of the mycobacterial respiratory chain as a target space for new antibiotic agents. Targeting simultaneously multiple respiratory enzyme complexes in *Mycobacterium tuberculosis* is regarded as one of the most effective options to treat TB, shorten drug administration regimes, and reduce the opportunity for the emergence of drug resistance^5,6^. In this light, new lines of evidence point to a crucial role of the cytochrome *bd* oxidase for mycobacterial pathophysiology^7–11^. This quinol-oxidizing terminal oxygen reductase is encoded by the *cydAB* gene cluster and is a central component of the branched respiratory chain of *M. tuberculosis*. The clinical relevance of the cytochrome *bd* oxidase is highlighted by deletion mutants that show dramatically increased susceptibility to the *bc*_1_:*aa*_3_ supercomplex inhibitor Q203^5,11^. This synergistic lethal interaction between the two respiratory branches of *M. tuberculosis* demonstrates that cytochrome *bd* oxidase is a major target for combating multi and extensive drug-resistant (MDR/XDR) tuberculosis infections^12^.

Previous structural insights into the cytochrome *bd* oxidase family have been limited to the first reported structure from *Geobacillus thermodenitrificans* (*G. th*) at 3.8 Å resolution (PDB: 5DOQ) and the recently published cryo-EM structures of the prototypic enzyme from *Escherichia coli* (*E. coli*)^13–15^ at 2.7–3.3 Å resolution (PDB: 6RKO, 6RX4). While the core architecture of these two known *bd* oxidase structures remains similar, structural details on the level of side chains, arrangement and composition of prosthetic groups, and the presence of accessory subunits differ substantially. Structural divergence and mechanistic diversity observed between different *bd* oxidase homologs thus emphasize the need for detailed knowledge of the atomic structure of the *bd* oxidase from *Mycobacterium tuberculosis*, in order to provide a solid framework for structure-guided drug design^13–15^. Here we present the cryo-EM structure of the *bd* oxidase from *M. tuberculosis* at 2.5 Å resolution which reveals a previously unknown MK-9 binding site at heme *b*_595_.

## Results

### General architecture and subunit composition

**Fig. 1:**
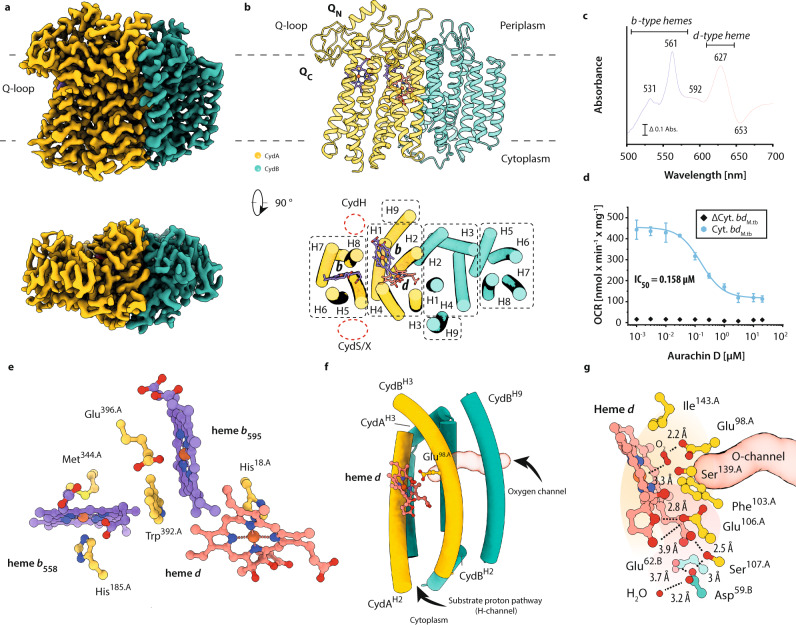
Cryo-EM structure of the *bd* oxidase from *M. tuberculosis*. **a** Surface representation of the *bd* oxidase cryo-EM density map at 2.5 Å resolution. The *bd* oxidase consists of a heterodimeric CydAB core dimer The Q-loop of CydA comprises a mostly disordered Q_N_ and an ordered Q_C_ segment that are both exposed to the periplasm. **b** Cyt. *bd*_*Mtb*_ contains 18 membrane-spanning helices. CydA and CydB form a pseudo-symmetric dimer. All heme cofactors (*b*_558_, *b*_595_, and heme *d*) are located within CydA. Dashed red circles indicate the positions of accessory transmembrane helices in other *bd* oxidase structures. **c** Redox-minus-oxidized difference UV–vis spectrum of purified cyt. *bd*_*Mtb*_. **d** Oxygen consumption rates from biological replicates (*n* = 7) of IMVs containing overproduced cytochrome *bd* oxidase. Control experiments were performed with IMVs from *ΔcydAB* cells containing an empty expression vector. Control data were obtained from biological triplicates (*n* = 3). All data are presented as mean values **±** SD. **e** Triangular arrangement of the heme cofactors in CydA. **f** The oxygen conducting channel emerging from the membrane plane and running towards the active site. **g** Heme *d* sits in an amphipathic pocket where the hydrophilic proton channel and hydrophobic oxygen pathway converge at the dioxygen binding site. Distances between water molecules and conserved hydrophilic residues are shown. Color scheme: CydA, yellow; CydB, green; *b*-type hemes, purple; heme *d*, coral.

The cytochrome *bd* oxidase from *M. tuberculosis* (cyt. *bd*_*Mtb*_) was recombinantly produced in a *Mycobacterium smegmatis* mc^2^ 155 Δ*cydAB* strain harboring a markerless deletion of its chromosomal cytochrome *bd* oxidase (Δ*cydAB*). Cofactor assembly and oxygen reductase activity were validated via UV–Vis spectroscopy and oxygen consumption measurements (Supplementary Fig. 1, Fig. 1c, d). The three-dimensional structure of POPC nanodisc reconstituted cyt. *bd*_*Mtb*_, in the as isolated mixed-valence state was determined by single-particle electron cryo-microscopy (cryo-EM) to a resolution of 2.5 Å (Supplementary Fig. 1b, Fig. 1a).

The cyt. *bd*_*Mtb*_ is structurally characterized by the presence of a pseudo-symmetrical heterodimer formed by CydA and CydB (Fig. 1). Each subunit is composed of nine transmembrane helices arranged in two four-helix bundles along with an additional peripheral helix (Fig. 1b, Supplementary Fig. 2). The CydA subunit represents the catalytically active component of the enzyme complex, harboring the three prosthetic heme groups, and the quinol binding and oxidation domain (Q-loop) (Fig. 1b). Binding of small molecules to this domain can efficiently inhibit access of substrate quinols to their oxidation site and thus diminish oxygen reductase activity^7,12,13,16^. Chromosomal deletion of the small CydX subunit encoded immediately downstream the *cydB* gene results in loss of active site high-spin heme groups of proteobacterial *bd* oxidases^17–19^. Mycobacterial genomes do not encode for CydX-like accessory subunits in their *bd* oxidase operons. We took into account that such small subunits could be encoded at different loci of mycobacterial chromosomes and searched for homologous sequences. Our results indicate the absence of orphan genes that encode for known accessory *bd* oxidase subunits in mycobacteria. Despite the close structural similarity to the cytochrome *bd* oxidase from *Escherichia coli* (cyt. *bd*_*Ecoli*_), the absence of a CydX subunit homolog neither affects cofactor assembly of cyt. *bd*_*Mtb*_, nor does it influence cofactor composition as shown by our cryo-EM structure and spectroscopic data. The reduced-minus-oxidized UV–visible difference spectrum confirms the presence of hemes *b*_558_, *b*_595_, and *d* (Fig. 1c). The spectral properties of these hemes closely match those previously reported for the cytochrome *bd* oxidases of *E. coli* and *Azotobacter vinelandii*^20–23^. While we cannot fully exclude the possibility of the existence of small accessory subunits of unknown sequence encoded by the genome of *M. tuberculosis*, we can confirm that the structural genes of the *bd* oxidase operon are sufficient for complete assembly of the canonical core dimer and the functional insertion of the prosthetic heme groups (Fig. 1b, Supplementary Fig. 4).

The triangular heme arrangement in cyt. *bd*_*Mtb*_ with its coordinating polypeptide framework and the oxygen entry channel of the mycobacterial enzyme are consistent with structures reported previously (Fig. 1e–g)^13,14^. Apart from these structural similarities, we noted several differences between the structure of cyt. *bd*_*Mtb*_ and those from Proteobacteria and Firmicutes. One of the most prominent differences is found in the architecture of the CydB subunit. The cavity that harbors a stabilizing UQ-8 molecule in cyt. *bd*_*E.coli*_ is absent in cyt. *bd*_*M.tb*_. Instead, we identify a series of Trp and Phe residues that occupy this space. They form stabilizing van-der-Waals contacts between TMHs 3, 4, 5, and 8 that render the structural quinone unnecessary (Supplementary Fig. 5). Such a stabilizing network of aromatic side chains is not present in the quinone-free CydB subunit of the S-subfamily cytochrome *bd* oxidase from *Geobacillus thermodenitrificans* (cyt. *bd*_*Gth*_). Lack of either stabilizing protein–protein or protein–quinone interactions in this unoccupied cavity may explain the high B-factors observed in the crystal structure of cyt. *bd*_*Gth*_. Despite fold similarity, the non-catalytic CydB subunits of cyt. *bd*_*Mtb*_, cyt. *bd*_*Ecoli*_, and cyt. *bd*_*Gth*_ show larger sequence variability between each other as compared to their respective CydA subunits. This suggests a weaker selection pressure as previously pointed out by distance analyses suggesting that *cydB* genes undergo accelerated evolutionary changes relative to *cydA* genes^24^.

### Solvent accessibility

**Fig. 2:**
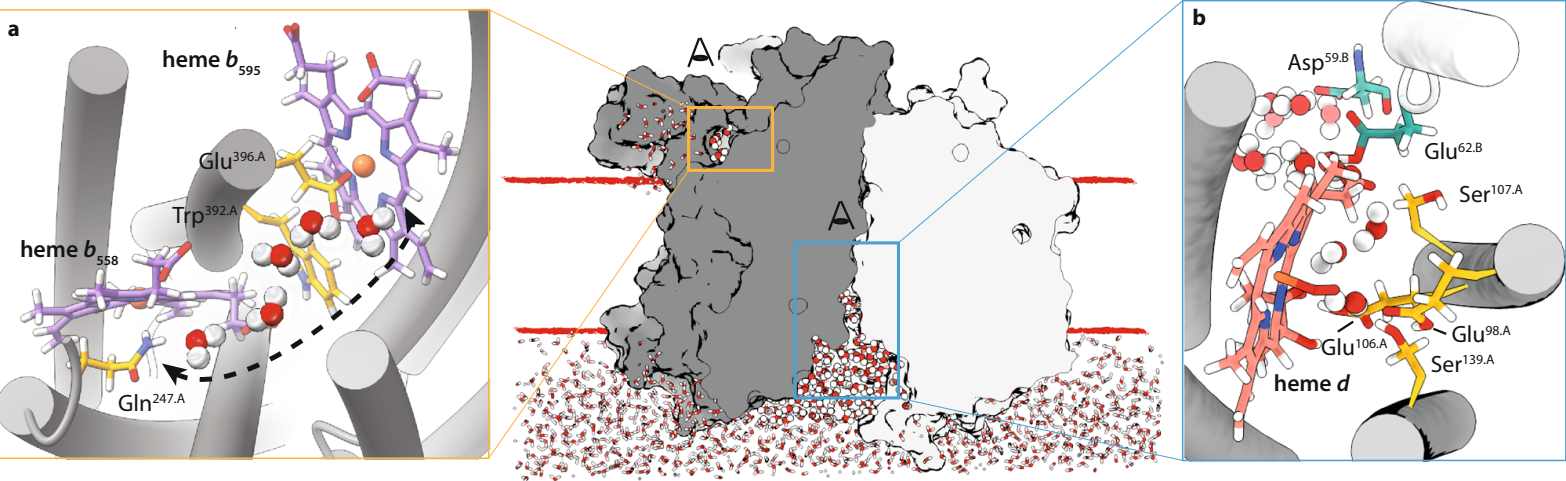
Molecular dynamics simulation of interior hydration. Representative snapshots of solvent molecules consistently identified in MD simulation runs. **a** The left panel shows the newly identified water chain between the b-type hemes in top view orientation. **b** The right panel shows the path of water molecules entering at the CydAB interface and running towards the oxygen reduction site. Residues lining the putative proton channel are indicated. CydA, gray; CydB, light grey; *b*-type hemes purple; heme *d*, coral. Water molecules are shown as tri-sphere units (O: red; H: white).

Based on the structural framework of cyt. *bd*_*Mtb*_, we performed atomistic molecular dynamics (MD) simulations in a POPC lipid environment to probe solvent accessibility and understand the dynamic behavior of water within these channels. Our data show the existence of a major water filled channel that starts as a shallow cavity on the cytoplasmic side with a diameter of 20 Å at the CydAB interface (Fig. 2, Supplementary Fig. 3). The cavity narrows to a 4 Å channel that runs perpendicular to the membrane plane between the inter-subunit four-helix bundle formed by TMH2/3.A and TMH2/3.B. This solvent-accessible channel is filled with water molecules that are abundant near the cytoplasmic surface and become less abundant as the channel extends towards Asp^59.B^. The hydrogen-bond network formed by Asp^59.B^, Glu^106.A^, Ser^107.A^, Ser^139.A^, and Glu^62.B^ connects the solvent channel to the oxygen reduction site, which suggests a role in proton delivery (Fig. 1g). On the periplasmic side of the membrane, between TMHs 5 and 6, we identify an entry site for water molecules running horizontally towards heme *b*_595_. Within the space between the two b-type hemes our simulation suggests the existence of a string of solvent molecules connecting the propionate groups of heme *b*_558_ and the side chain of Glu^396.A^ over a distance of 12 Å (Fig. 2, Supplementary Fig. 3a). The functional role of this solvent-filled region can be attributed to a “dielectric well”, similar to what has been described for the function of water molecules within the H-channel of A-type cytochrome *c* oxidases (C*c*Os)^25^. In this structural context solvent molecules could either facilitate charge compensation via protonation and deprotonation of carboxyl groups, or by reorientation of dipole charges in response to redox conditions of the prosthetic heme groups. Localization of most of the described waters molecules is consistent between structural and simulation data.

### The mycobacterial *bd* oxidase exhibits a unique Q-loop architecture

**Fig. 3:**
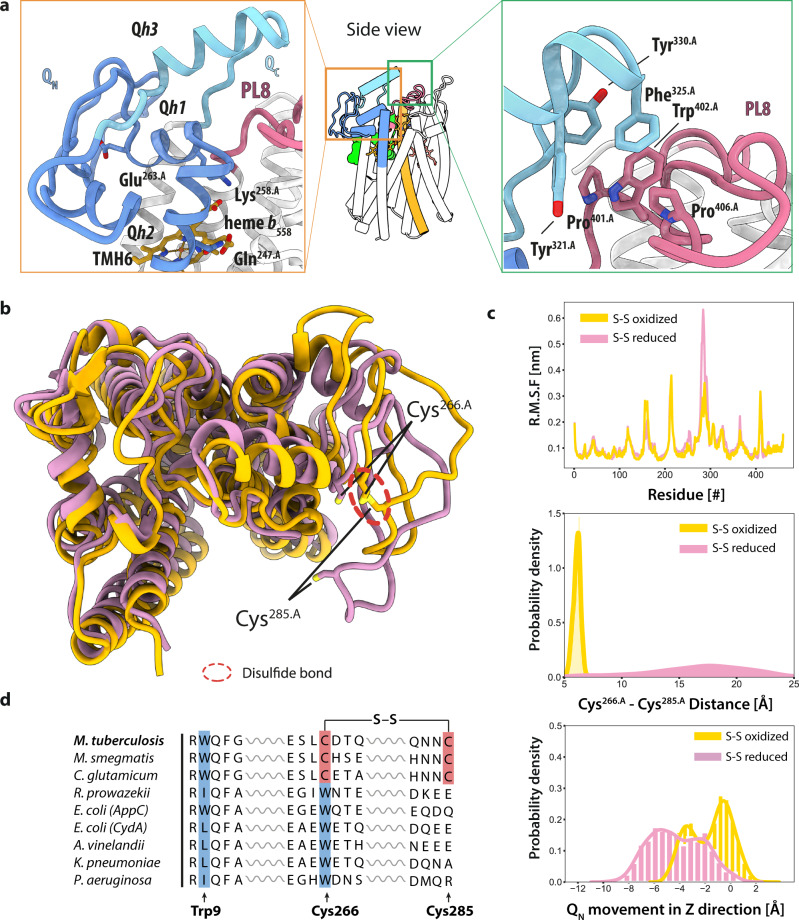
Q-loop architecture of cyt. *bd*_*Mtb*_. **a** Left: The mycobacterial Q-loop is organized in a mostly disordered Q_N_ (blue) and a rigid, well-ordered Q_C_ segment (cyan). Q_N_ starts at Pro^256.A^ and contains the horizontal helix Q*h1* with the conserved residues Lys^258.A^ and Glu^263.A^ that covers heme *b*_558_, and a second small helix Q*h2* comprising residues Tyr^296.A^ to Ala^303.A^. The Q_C_ segment is exposed to the periplasm and consists of the third Q-loop helix Q*h3* that covers the surface of the TMHs 6 and 7. Right: Van-der-Waals interaction cluster between Q*h3* and the periplasmic loop 8 (PL8) of CydA. **b** Top view of a superposition of CydA snapshots obtained from MD simulations performed in presence and absence of the Cys^266.A^-Cys^285.A^ disulfide bond. Positions of cysteine residues are indicated. The red dashed circle indicates the location of the intact disulfide bond. CydA (oxidized), yellow; CydA (reduced), violet. **c** Top: average root mean square fluctuation plot of CydA residues in MD simulations for 2 × 750 ns with reduced and oxidized disulfide bond (Cys^266.A^-Cys^285.A^) of the Q_N_-loop. Middle: Data plots of C_α_-C_α_ distance distribution between disulfide-forming cysteines during MD simulations. Bottom: Distribution of Q_N_ displacement in *Z* direction during MD simulations. Yellow data plots indicate a simulation setup with intact disulfide bond. Purple data points indicate a simulation setup with a reduced disulfide bond. **d** Sequence alignment of representative cytochrome *bd* oxidases.

Cytochrome *bd* oxidases are classified into either a short (S) or a long (L) subfamily depending on the length of the Q-loop domain^20^. Members of the L-subfamily exhibit a C-terminal insertion in their Q-loop which adopts a helix-turn-helix fold located at the periplasmic surface of CydA. This C-terminal half of the Q-loop is essential for folding and stability of the CydA subunits of the L-subfamily *bd* oxidases^26,27^. Several homologs, including the one from *M. tuberculosis*, cannot be clearly assigned to either of these two subfamilies (Supplementary Fig. 6). Our cryo-EM structure provides structural insight into this unclassified group of cytochrome *bd* oxidases. In the structure of cyt. *bd*_*Mtb*_, the well-conserved N-terminal segment of the Q-loop (Q_N_) is completely resolved and comprises residues Pro^256.A^ to Val^309.A^ (Figs. 1b, 3a). This segment emerges between TMH6 and TMH7 of CydA and is characterized by two short helical motifs (Q*h1*, Q*h2*) and a large disordered loop region (Fig. 3a). The Q*h1* helix plays a critical role in coordinating propionate A of heme *b*_558_ and substrate quinols^16^.

The C-terminal stretch (residues Thr^310.A^ to Asn^333.A^) of the mycobacterial Q-loop (Q_C_) contains a short horizontal helix Q*h*3 which is exposed to the periplasm (Fig. 3a). It does not span the entire periplasmic surface of the CydA subunit as does the structurally similar but sequence unrelated horizontal helix of cyt. *bd*_*Ecoli*_. In the mycobacterial enzyme this helix covers the periplasmic surface of TMHs 6 and 7 and extends only up to the large periplasmic loop connecting TMHs 8 and 9 (PL8) (Fig. 3a). At this interface, a hydrophobic cluster is located which is composed of aromatic residues Tyr^321.A^, Phe^325.A^, Tyr^330.A^ of Q*h3*, and the conserved Pro^401.A^, Trp^402.A^, and Pro^406.A^ residues from PL8 (Fig. 3a). The aromatic residues Phe^325.A^ and Tyr^330.A^ are known to be critical for the activity of mycobacterial cytochrome *bd* oxidases^28^. The location of these residues suggests that they play a structural role by stabilizing the Q*h3*-PL8 interaction instead of being involved in substrate recognition or redox chemistry of the oxidase. The C-terminal half of PL8, which represents a short polypeptide insertion unique for the mycobacterial *bd* oxidases, forms a series of backbone hydrogen bonds with the N-terminal segment of PL8 (Supplementary Figs. 6, 7). Analysis of atomic interactions at the Q_C_–PL8 interface showed that nine hydrogen bonds of PL8 (segments 399–418) were preserved during simulation runs. Further, our data showed that π-π interactions between Tyr^321.A^, Phe^325.A^, Tyr^330.A^ of Q*h3* and Trp^402.A^ of PL8, hydrophobic interaction between Phe^325.A^ and Pro^401.A^, as well as the cation- π interaction between Phe^325.A^ and Arg^415.A^ remained stable for the entire duration of the runs. Overall, our data demonstrate that this stabilizing network contributes to the rigidity of PL8 important for maintaining the interaction with Q*h3*. Our structural findings confirm that the Q-loop architecture of the mycobacterial cytochrome *bd* oxidases resembles that of the large Q-loop variants. Although the length of the horizontal Q*h3* helix is shorter compared to the analogous helix of L-subfamily members, its placement and interaction pattern with the CydA surface suggest that it has a similar stabilizing effect (Supplementary Fig. 4b)^28^.

A distinguishing feature of the mycobacterial Q-loop is the presence of a disulfide bond between Cys^266.A^ and Cys^285.A^ within the Q_N_ region exposed towards the periplasmic space (Fig. 3b). Our simulations demonstrate that the disulfide bond rigidifies the disordered Q-loop segment and reduces flexibility between Q_N_ and Q*h1* (Fig. 3c). This structural property is in stark contrast to the Q_N_ domain of cyt. *bd*_*Ecoli*_ which does not contain a disulfide at this position (Fig. 3d)^13,14^. For this enzyme, two independent structural studies confirmed that even in the presence of bound inhibitors the characterized binding site located at Q_N_-loop remains highly flexible and does not adopt a distinct conformation^13,14,16^. The dynamic nature of the proteobacterial Q_N_ is most likely required for a transient binding of substrate quinols in order to maintain continuous electron flow and oxygen consumption of the high-turnover *bd* oxidase.

In order to characterize the binding of competitive quinol-like inhibitors to the mycobacterial Q-loop, we determined cyt. *bd*_*Mtb*_ cryo-EM structures in the presence of Aurachin D and AD3-11 at 3.3 Å and 2.7 Å resolution, respectively (Supplementary Fig. 1d). Even with a molar excess of 50–100:1 of these highly potent inhibitors, we were not able to identify any density consistent with bound quinolone derivatives at the conserved and previously characterized binding site located at Q_N_^13,16^. This observation agrees with data from our MD simulations showing that both reduced and oxidized MK-9 do not form specific interactions at the Q_N_ domain and migrates into the membrane space (Supplementary Fig. 10). We conclude that the confined conformation of the Q_N_-region sterically hinders an interaction between substrate molecules and the highly conserved Glu^263.A^ which is involved in quinol binding and oxidation in immediate proximity to heme *b*_558_^16^.

### Identification of a MK-9 binding pocket

**Fig. 4:**
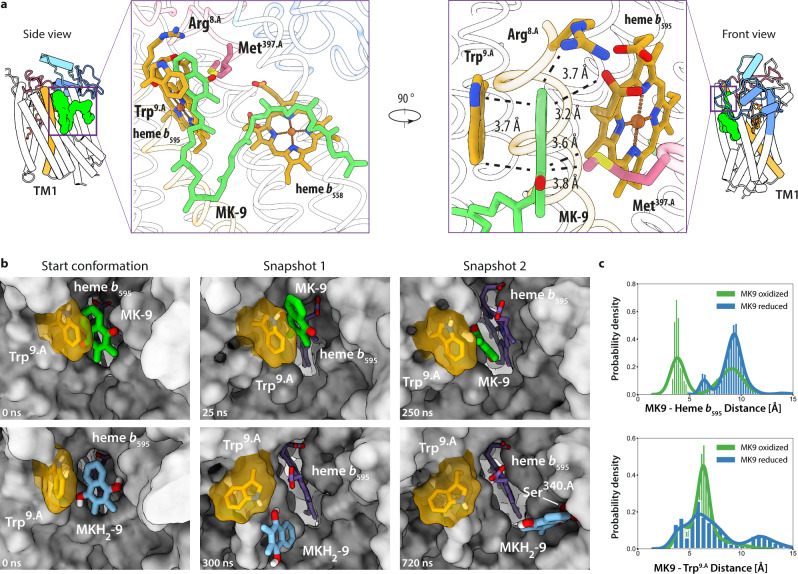
Allosteric MK-9 binding site of cyt. *bd*_*Mtb*_. **a** Left: Side view closeup of the MK-9 binding pocket composed of residues from TMH1, TMH9, and the heme *b*_595_ porphyrin scaffold. Right: Front view closeup of the MK-9 binding pocket composed of residues from TMH1, TMH9, and the heme *b*_595_ porphyrin scaffold. Van-der-Waals interaction distances are indicated. **b** Representative snapshots of different MK-9 binding modes during 2 × 750 ns MD simulations in reduced and oxidized states. For simplicity, hydrocarbon chains of MK-9 molecules are not shown. **c** Distance histograms between MK-9 and heme *b*_595_ (Top), and MK-9 and Trp^9.A^ (Bottom) during MD simulations. Blue data plots indicate a simulation setup with reduced MK-9. Green data plots indicate a simulation setup with oxidized MK-9. Oxidized MK-9, green; reduced MK-9, blue; heme *b*_595_, purple; Trp^9.A^, yellow.

In proximity to the disulfide bond, within the cleft formed between TMH 1 and 9 we identified a bound menaquinone-9 (MK-9) molecule (Fig. 4a). LC-MS analysis confirmed that this co-purified MK-9 group is in the oxidized state (Supplementary Fig. 8). The local environment that forms this previously unknown MK-9 binding pocket is composed of the heme *b*_595_ porphyrin scaffold, Arg^8.A^ and Trp^9.A^ of TMH1, and Met^397.A^ of TMH 9 (Fig. 4a). The Trp^9.A^ side chain represents the main contributor to the cavity formation and shields the MK-9 head group from the surrounding membrane environment. Its aromatic plane is aligned parallel to the naphthoquinone head group of MK-9 with geometry and distance consistent with stacking π–π interactions (Fig. 4a). On the distal side of the naphthoquinone group Met^397.A^ is in close distance to form stabilizing van-der-Waals interactions (Fig. 4a). Remarkably, this quinone binding site is occupied by the CydH subunit in cyt. *bd*_*Ecoli*_. We characterized the binding dynamics of the identified MK-9 molecule in the reduced and oxidized state via MD simulations (Fig. 4b, c). In the oxidized setup, MK-9 remained bound to its specific pocket by either forming π–π interactions with Trp^9.A^ (Fig. 4b—snapshot 1) or heme *b*_595_ (Fig. 4b—snapshot 2). Oxidized MK-9 intermittently forms H-bonds with both Arg^8.A^ and Trp^9.A^ (10–25% of the simulation time). In the reduced state, the menaquinol head group shows increased dynamics and transiently adopts different binding positions between TMH1 and 9 while the interaction with Trp^9.A^ is maintained. Analysis of H-bonds shows that during 20% of the simulation time reduced MK-9 forms an intermittent H-bond with the propionate group of heme *b*_595_ (Fig. 4b snapshot 1). In 15% of the simulation time, MK-9 instead forms a H-bond with Ser^340.A^ after flipping to the other side of heme b_595_ towards TMH8 (Fig. 4b—snapshot 2).

## Discussion

Given the two remarkable structural features identified in the cyt. *bd*_*Mtb*_ structure namely the disulfide within the Q-loop and the occurrence of the MK-9-binding site near the location of the *b*_595_ heme, the question arises whether their existence is exclusively conserved among mycobacterial cytochrome *bd* oxidases. If functional association imposes selective constraints, then these two structural properties would evolve in a correlated fashion. In this regard, a multiple sequence alignment was generated using 561 CydA sequences from the manually curated representative genome list (Seed) of the protein family database (Pfam). We found that 94 CydA sequences (16.8%) exhibited a conserved tryptophan residue in TMH1 at this position (Supplementary Fig. 9a). More than half of these sequences (*n* = 58) belong to the phylum of Actinobacteria that includes the important pathogens from genera of Mycobacteria, Nocardia, and Corynebacteria. Among the total number of analyzed sequences only 30 orthologs (5.7%) exhibited the two cysteine residues participating in disulfide bond formation within the Q_N_-loop (Supplementary Fig. 9b). Intriguingly, these sequences all belong to members of the phylum of Actinobacteria and also show the presence of the signature Trp^9.A^ residue in TMH1 (Supplementary Fig. 9b, Fig. 3d). The disulfide bond confined Q-loop domain thus appears to have a strong selective pressure for the Trp^9.A^ residue, and consequently infers the existence of the identified MK-9 binding site.

**Fig. 5:**
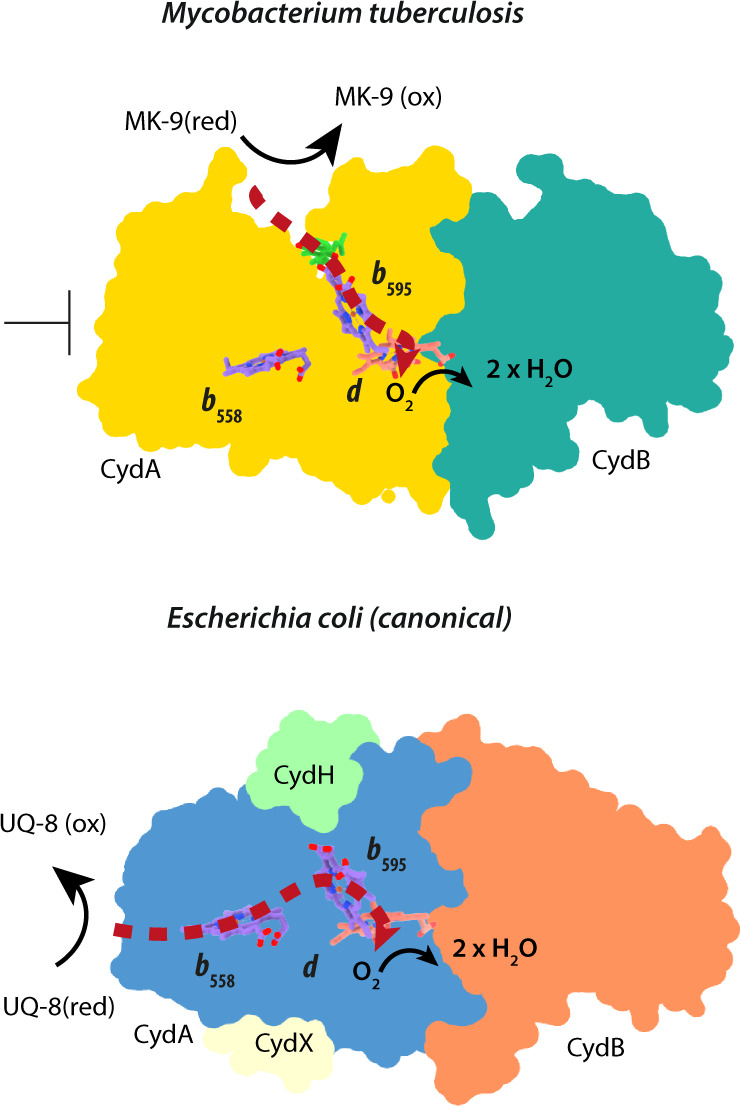
Schematic overview of subunit composition and putative electron transfer pathways of cyt. *bd*_*Mtb*_*and* cyt. *bd*_*Ecoli*_. Red dashed lines illustrate proposed electron transfer routes. Subunits are indicated by different colors.

Judging from its peripheral location and close distance to heme *b*_595_, it is unlikely that the MK-9 molecule acts solely as a structural component contributing to stability of the polypeptide framework. In cyt. *bd*_*Mtb*_ the existence of the MK-9 binding site in immediate proximity of heme *b*_595_ strongly suggests a function as a quinol oxidation domain compensating for the inability of the mycobacterial Q-loop to bind substrate. Membrane quinols equilibrating within the cleft formed by TMHs 1 and 9, as observed in our simulations, could transfer electrons onto heme *b*_595_ bypassing an initial electron transfer onto heme *b*_558_ (Fig. 5). The presence of the disulfide bond in the Q_N_ raises the question of whether the activity of the *bd* oxidase in *M. tuberculosis* can be differentially regulated by breaking or forming the disulfide bond through which the canonical electron transfer pathway would be re-activated.

The molecular structure of the *bd* oxidase from *M. tuberculosis* provides the long-sought atomic framework for the design of highly specific drugs to target this respiratory enzyme. It may be possible to design inhibitors that prevent the interaction between the Q_C_ domain and PL8 and thus interfere with the assembly of a functional enzyme. An alternative target is the membrane-embedded entry site of the oxygen-conducting O-channel that would be targeted by hydrophobic compounds to block the channel and abolish oxygen uptake. The newly identified secondary MK-9 binding site represents another highly specific target site. The latter however requires more in-depth insights about the detailed mechanistic role of this unique domain.

## Methods

### Generation of the cyd*ABDC* expression construct for cytochrome *bd* oxidase production

To create the expression construct for production of cytochrome *bd* oxidase, the *cydABDC* operon from *Mycobacterium tuberculosis* was cloned into the episomal expression vector pYUB28b^29^. For affinity purification, the 3′ end of *cydB* gene (C-terminus of CydB) was modified with a TEV cleavage site sequence followed by a FLAG affinity tag sequence. Initially, *cydAB* was amplified from *M. tuberculosis* genomic DNA using the primer pair cydAB_fw (5′-TAAGAAGGAGATATACCATGAATGTCGTCGACATTTCGCGGTG-3′) and cydAB_rv1 (5′-GCCAGACCGGTGGGTGGAGGTAT-3′). Following this, two more rounds of PCR amplification were performed on the *cydAB* PCR product using different reverse primers in combination with cydAB_fw. Firstly, the TEV cleavage site was added with cydAB_rv2 (5′-GCCCTGAAAATACAGGTTTTCGGGCGCGCGCCTCGCCAGACCGGTGGGTGGAGG -3′) and then the FLAG tag was added with cydAB_rv3 (5′- GCCTTGGTGCTCACTTATCGTCGTCATCCTTGTAATCGCCCTGAAAATACAGGTTTTC-3’) to give the final *cydAB* PCR product. The *cydDC* portion of the operon was amplified using the primer pair cydDC_fw (5′- CGATAAGTGAGCACCAAGGCCCGGGGAACC-3′) and cydDC_rv (5′- TCGAGTGCGGCCGCAAGCTTTCAGGTCTTTGCGCTGGCGTT-3′). The final *cydAB* and *cydDC* PCR products were ligated into pYUB28b using NEBuilder^®^ HiFi DNA Assembly as per the manufacturer’s instructions. The resulting mix was then transformed into *E. coli* MC1061^30^. Initial screening of the resulting colonies was done using PCR with the primer pair cydAB_fw and cydDC_rv. Positive clones were confirmed using Sanger sequencing. The confirmed *cydABDC* expression construct was named pLHcyd. All restriction enzymes and DNA modifying enzymes were used from New England Biolabs. All constructs were confirmed using either PCR or restriction digestion followed by DNA sequencing. PCR for construct creation was done using Thermo Fisher Phusion polymerase following the recommended conditions with appropriate annealing temperatures and extension times.

### Production of cytochrome *bd* oxidase from *M. tuberculosis*

The cytochrome *bd* oxidase from *M. tuberculosis* (*M. tb)* was produced in *Mycobacterium smegmatis* mc^2^ 155 Δ*cydAB* cells transformed with the pLHcyd plasmid carrying genes of the cytochrome *bd* oxidase from *M. tb* with a C-terminal FLAG-tag modification at *cydB*, the CydDC assembly factor of the cytochrome *bd* oxidase from *M. tb*, and a hygromycin resistance cassette^10^. Mycobacteria were grown in LB Lennox media (10 g/L tryptone, 5 g/L yeast extract and 5 g/L NaCl) with 0.05% w/v Tween 80 and 50 µg/ml hygromycin B added before use. Single aliquots of electrocompetent cells were transformed with 1 µl DNA and 260 µl of 10% ice-cold glycerol. Electroporation was carried out in 0.2 cm cuvettes using a Bio-Rad Gene Pulser: *R*  = 1000 Ω, *Q*  = 25 µF and *V*  = 2.5 kV. Cells were immediately resuspended in 1 ml LBT media and incubated for 3 h at 37 °C with constant shaking at 220 rpm. Positive transformants were selected by plating cells on LBT agar containing 50 µg/ml hygromycin B.

For production of cytochrome *bd* oxidase, a single colony of the production strain (see above) was used to inoculate a pre-culture in LBT media containing 50 µg/ml hygromycin B. This culture was grown for 72 h at 37 °C and 200 rpm. The pre-culture was used to inoculate the production culture with a 1:100 dilution. Cytochrome *bd* oxidase production was carried out for 72 h at 37 °C with constant shaking at 200 rpm to obtain maximal protein production. Cells were harvested by centrifugation at 4000 × *g* for 20 min at 4 °C and flash frozen in liquid nitrogen before storage at −80 °C.

All steps of the purification process were carried out at 4 °C. Cells were resuspended in buffer containing 50 mM Tris-HCl (pH 7.4), 5 mM magnesium chloride, 0.05% Tween 80 and 0.1 mg/ml Pefabloc SC (5 ml buffer/1 g cells). Cells were disrupted using a high-pressure homogenizer (Avestin Emulsiflex C3) via several passages (4–6) at 22,000 psi. The lysate was centrifuged at 10,000 × *g* for 20 min to remove cell debris and unlysed cells. The supernatant of the low-speed centrifugation step was subsequently centrifuged at 225,000 × *g* for 90 min. Pelleted membranes were resuspended in storage buffer [20 mM Tris-HCl (pH 7.4), 0.05% Tween 80 and 10% glycerol]. Prior to freezing the protein concentration of the isolated membrane fraction was adjusted to 10 mg/ml using Bradford reagent.

### Purification of cytochrome *bd* oxidase from *M. tuberculosis*

Isolated membranes were solubilized with 1% dodecyl-ß-D-maltoside (DDM) in a mass ratio of 1 mg detergent per 5 mg of membrane protein for 100 min at 4 °C. The *bd* oxidase was purified via FLAG tag affinity chromatography using an ANTI-FLAG^®^ M2 affinity matrix (Merck- Millipore). For column conditioning and the washing step during purification (5 column volumes [CV]) a buffer containing 50 mM Tris-HCl (pH 7.4), 150 mM NaCl, and 0.02 % DDM was used (buffer A). For step elution (5 × 1 CV), buffer A was supplemented with FLAG peptide (Merck- Millipore) to a final concentration of 0.1 mg/ml (buffer B). For sample polishing, size exclusion chromatography (SEC) was performed using a Superdex 200 10/300 Increase column. The SEC running buffer contained 20 mM Tris-HCl (pH 7.4), 150 mM NaCl, and 0.02% DDM.

### Production and purification of MSP1D1

MSP1D1 production was carried out according to published protocols^31^. In brief, Bl21Gold (DE3) cells transformed with pMSP1D1 were grown in LB-Kan medium (50 µg/ml kanamycin) at 37 °C until an OD_600_ of 0.6 was reached before MSP1D1 production was induced by addition of IPTG to a final concentration of 1 mM. Growth was carried out for 4 h before cell harvest. MSP1D1 was purified according to standard conditions described earlier^31^ and stored in a buffer containing 20 mM Tris-HCl (pH 7.5) and 150 mM NaCl.

### Reconstitution of *bd* oxidase in lipid nanodiscs

Cytochrome *bd* oxidase was reconstituted into lipid nanodiscs according to protocols by Ritchie et al. ^31^. In brief, POPC, MSP1D1, and *bd* oxidase were mixed in a molar ratio of 400:10:1 and incubated at 4 °C for 1 h. Detergent was removed from the mixture by sequential addition of 4 ×0.2 mg dried Bio-Beads every 2 h. Finally, the mixture was incubated at 4 °C for additional 12 h while shaking. Bio-Beads were removed via filtration and the reconstituted *bd* oxidase particles were separated from empty nanodiscs via SEC on a Superdex 200 10/300 Increase column using a buffer containing 20 mM Na-HEPES (pH 7.5) and 100 mM NaCl. Nanodisc reconstituted samples were used for downstream cryo-EM experiments. Aurachin D was added to the detergent-solubilized protein solution before reconstitution at a final concentration of 500 µM. AD3-11 was added to the detergent DDM solubilized protein solution before reconstitution at a final concentration of 1 mM. Inhibitor concentration was maintained throughout the reconstitution procedure and until grid preparation.

### Sample vitrification

Quantifoil R1.2/1.3 copper grids (mesh 200) were washed in chloroform and subsequently glow discharged twice with a PELCO easiGlow device at 15 mA for 45 s. A volume of 4 µl sample (1.5 mg/ml) was applied on a grid immediately before plunge freezing. Samples were vitrified at 4 °C, 100% humidity, and a blot force of 20 using a Vitrobot IV device (Thermo Fischer). Blotting was carried out for 4 s before plunge freezing in liquid ethane.

### Cryo-EM image recording

A total of 12070 movies were recorded in Energy-Filtered Transmission Electron Microscopy mode using a Titan Krios G3i microscope operated at 300 kV (Thermo Fisher). Electron-optical alignments were adjusted with EPU 2.9. Micrographs were recorded using automation strategies of EPU 2.9 in electron counting mode with a Gatan K3 direct electron detector at a nominal magnification of ×105,000, corresponding to a calibrated pixel size of 0.837 Å. Dose fractionation movies were recorded at an electron flux of 15 e^-^ x pixel^−1^ × s^−1^ for 5 s, corresponding to a total dose of ~108 e^-^/A^2^. Images were recorded at 1.1–2.1 µm under focus.

### Cryo-EM image processing

MotionCor2 was used to correct for beam-induced motion and to generate dose-weighted images^32^. CTFfind was used to determine the contrast transfer function (CTF) parameters and perform correction steps^33^. Images with estimated poor resolution (>4 Å) and severe astigmatism (>300) were removed at this step. Particles were picked by crYOLO^34^. A total of ca. 15 M particles were picked and used for all further processing steps. Initial model building, 3D classification, CTF refinement, Bayesian polishing, ND1D1 volume subtraction, and final map reconstructions were performed with RELION-3.1^35^. A detailed overview of our processing workflow is given in SI.

### Model building and refinement

The atomic model of the cytochrome *bd* oxidase was built in Coot (version 0.8.9) initially using the pdb model of the cytochrome bd-I oxidase from *E. coli* (6RKO) as reference structure^36^. After manual backbone tracing and docking of side chains in the respective map densities, we performed real-space refinement in Phenix (version 1.14)^37^. Refinement results were manually inspected and corrected if needed. The finalized model was validated by the MolProbity online server^38^. Map-to-model cross validation was performed in Phenix (version 1.14). FSC_0.5_ was used as cutoff to define resolution. A summary of the model parameters and the corresponding cryo-EM map statistics is found in Supplementary Table 1. The finalized model was visualized using ChimeraX^39^.

### Analysis of tunnels and interior cavities

Tunnels and interior cavities were mapped with MOLE 2.5 (bottleneck radius: 1.4 Å, bottleneck tolerance 3 Å, origin radius 5 Å, surface radius 8 Å, probe radius 5 Å, interior threshold 1.1 Å)^40^, considering densities for non-proteinogenic cofactors.

### Structural alignments

The overall folds of the mycobacterial *bd* oxidase subunits CydA and CydB were compared with corresponding subunit architectures of the available cytochrome *bd* oxidase structures from *E. coli* (6RKO, 6RX4) and *G.th* (5DOQ). The DALI protein database comparison sever was used for performing structure alignments and calculating Cα R.M.S.D values, and *Z*-scores^41^.

### Molecular dynamics simulation

The cyt. *bd*_*Mtb*_ structure was embedded in a bilayer composed of POPC using CHARMM-GUI^42^. All systems were hydrated with 150 mM NaCl electrolyte. The all-atom CHARMM36m force field was used for lipids, ions, cofactors, and protein with TIP3P water^43–47^. The heme *b* and O_2_ parameters were taken from the CHARMM36m, while heme *d* parameters were optimized using the FFTK toolkit^48^ based on the heme *b* parameters. MK-9 was parameterized in both reduced and oxidized forms using the FFTK toolkit. MD trajectories were analyzed using Visual Molecular Dynamics software^49^ and MDAnalysis package^50^. All simulations were performed using GROMACS 2019.6^51^. Starting systems were energy-minimized for 5000 steepest descent steps and equilibrated initially for 500 ps of MD in a canonical (NVT) ensemble and later for 7,5 ns in an isothermal–isobaric (NPT) ensemble under periodic boundary conditions. During equilibration, the restraints on the positions of nonhydrogen protein atoms of initially 4000 kJ mol^−1^ nm^2^ were gradually released. Particle-mesh Ewald summation^52^ with cubic interpolation and a 0.12-nm grid spacing was used to treat long-range electrostatic interactions. The time step was initially 1 fs, and was increased to 2 fs during the NPT equilibration. The LINCS algorithm was used to fix all bond lengths^53^. Constant temperature was set initially with a Berendsen thermostat, combined with a coupling constant of 1.0 ps. A semi-isotropic Berendsen barostat was used to set a pressure of 1 bar^54^. During production runs, the Berendsen thermostat and barostat were replaced by a Nosé–Hoover thermostat and a Parrinello–Rahman barostat^55,56^. Analysis was carried out on unconstrained simulations. Simulations of oxidized and reduced states of MK-9 and reduced Q-loop disulfide were performed for 2 × 750 ns.

### Mass spectrometry

Menaquinone-9 was identified in purified cyt. *bd*_*Mtb*_ samples by high-resolution mass spectrometry using a Shimadzu LCMS-9030 instument (Shimadzu Scientific Instruments Inc.). A volume of 2 µL of purified cyt. *bd*_*Mtb*_ was applied onto a C18 Shimadzu 2.0 × 50 mm Shim-pak XR-ODSIII column (1.6 µm) connected to a Shimadzu Nexera X2 HPLC system. The HPLC run was performed using 100 % methanol for isocratic elution. ESI source operating conditions: nebulizer gas 3 L/min, dry gas 10 L/min, interface voltage 3.00 kV, interface temperature 300 °C. Mass spectra were processed using LabSolutions Insight Explore LCMS software (Version 5.96). Oxidized menaquinone-9 was identified, as adducts, at a retention time of 37 min using a high-resolution fit. M^+^ H^+^ at 785.62571 Da (predicted 785.62311 Da), M^+^ Na^+^ at 809.62349 Da (predicted 809.62070 Da) M^+^ K^+^ 825.59962 Da (predicted 825.59464).

### Ultraviolet–visible (UV–Vis) absorption spectroscopy

Heme cofactors of the cytochrome *bd* oxidase were analyzed using ultraviolet-visible (UV–Vis) absorption spectroscopy. Spectra shown in this work were recorded with a Lambda 35 UV–Vis spectrometer from Perkin Elmer (Waltham, USA) using quartz micro-cuvettes from Hellma (Müllheim, Germany) with an optical path length of 10 mm. A sample volume of 400 μl was used for each measurement. UV–Vis data were collected in a spectral range between 380 nm and 700 nm with a scanning speed of 120 nm/min, data intervals of 0.2 nm, and an optical slit width of 1 nm. Samples were reduced by addition of sodium dithionite, and oxidized by addition of potassium ferrocyanide.

### Oxygen consumption in inverted membranes vesicles (IMVs)

IMVs of the indicated strains were prepared using a cell lysis protocol as described in the purification section. Oxygen consumption was measured using an Oroboros O2k fluorespirometer as previously described^11^. Briefly, IMVs were resuspended in buffer (50 mM Tris, 100 mM KCl, 5 mM MgCl_2_, pH 7.5) and 100 mM NADH was used to initiate respiration. For Aurachin D titrations, 500 nM TB47 (an inhibitor of cytochrome *bc*_1_:*aa*_3_ complex^10^) was added prior to the experiment to eliminate the contribution of the HCO type terminal oxidase to oxygen consumption. Membranes were used at a protein concentration 12.5  g/mL. Stepwise titrations were performed using the Oroboros O2k TIP2k automatic injection micropump, with rates measured at 120 s intervals between each injection. Data are normalized to protein concentrations that were estimated by BCA assay (Thermo), using a BSA standard. Addition of the *bc*_1_:*aa*_3_ supercomplex inhibitor TB47 allowed us to measure specific oxygen consumption rates of the cytochrome *bd* oxidase of IMV containing overproduced cytochrome *bd* oxidase. For negative control measurements, we used the IMVs of a *Mycobacterium smegmatis* mc^2^ 155 Δ*cydABDC* strain transformed with an empty pYUB28b vector. IC_50_ values were determined from logistic regression fits using Origin Lab Pro 9.5 (Additive GmbH, Germany). Samples were measured as biological replicates (*n* = 7) and control data was obtained from biological triplicates (*n* = 3).

### Multiple sequence alignments and taxonomy analyses

For conservation analysis of structural properties, we retrieved 561 CydA sequences from the manually curated representative genome list (Seed) of the protein family database (Pfam). Multiple sequence alignment was performed using Clustal Omega^57^. After manual curation, phylogenetic trees of orthologs containing either Trp^9.A^ or the cysteine pair in the Q_C_-loop domain were generated using NCBI Common Tree and visualized by FigTree (Version 1.4.4).

### Reporting summary

Further information on research design is available in the Nature Research Reporting Summary linked to this article.

## Supplementary information

Supplementary Information

Reporting summary

## Data Availability

The Cryo-EM maps of the cytochrome *bd* oxidase from *M. tuberculosis* are deposited at the Electron Microscopy Data Bank under accession numbers EMD-12451, EMD-12532, EMD-12533. The model of the cytochrome *bd* oxidase structure was submitted to the PDB with accession number 7NKZ. All other data are presented in the main text or Supplementary Information. Source data are provided with this paper.

## Source data

Source Data

## References

[1] World Health Organization, *Global Tuberculosis Report 2019* (https://www.who.int/teams/global-tuberculosis-programme/tb-reports/global-report-2019).

[2] Hards K (2015). Bactericidal mode of action of bedaquiline. J. Antimicrob. Chemother..

[3] Preiss L (2015). Structure of the mycobacterial ATP synthase F_o_ rotor ring in complex with the anti-TB drug bedaquiline. Sci. Adv..

[4] Andries K (2005). A diarylquinoline drug active on the ATP synthase of mycobacterium tuberculosis. Science.

[5] Kalia NP (2017). Exploiting the synthetic lethality between terminal respiratory oxidases to kill Mycobacterium tuberculosis and clear host infection. Proc. Natl Acad. Sci. USA..

[6] Berney M, Hartman TE, William R Jacobs J (2014). A mycobacterium tuberculosis cytochrome bd oxidase mutant is hypersensitive to bedaquiline. mBio.

[7] Lu P (2015). The cytochrome bd-type quinol oxidase is important for survival of Mycobacterium smegmatis under peroxide and antibiotic-induced stress. Sci. Rep..

[8] Bald D (2017). Targeting energy metabolism in Mycobacterium tuberculosis, a new paradigm in antimycobacterial drug discovery. mBio.

[9] Mascolo L, Bald D (2020). Cytochrome bd in Mycobacterium tuberculosis: a respiratory chain protein involved in the defense against antibacterials. Prog. Biophys. Mol. Biol..

[10] Lu X (2018). Pyrazolo[1,5-a]pyridine inhibitor of the respiratory cytochrome bcc complex for the treatment of drug-resistant tuberculosis. ACS Infect. Dis..

[11] Lee BS (2021). Dual inhibition of the terminal oxidases eradicates antibiotic‐tolerant Mycobacterium tuberculosis. EMBO Mol. Med..

[12] Lu P (2018). The anti-mycobacterial activity of the cytochrome bcc inhibitor Q203 can be enhanced by small-molecule inhibition of cytochrome bd. Sci. Rep..

[13] Safarian S (2019). Active site rearrangement and structural divergence in prokaryotic respiratory oxidases. Science.

[14] Theßeling A (2019). Homologous bd oxidases share the same architecture but differ in mechanism. Nat. Commun..

[15] Safarian S (2016). Structure of a bd oxidase indicates similar mechanisms for membrane-integrated oxygen reductases. Science.

[16] Tatsushi Mogi (2006). Probing the ubiquinol-binding site in cytochrome bd by site-directed mutagenesis†. Biochemistry.

[17] Chen H (2015). Evidence for the requirement of CydX in function but not assembly of the cytochrome bd oxidase in Shewanella oneidensis. Biochim. Biophys. Acta.

[18] Hoeser J (2014). Subunit CydX of Escherichia coli cytochrome bd ubiquinol oxidase is essential for assembly and stability of the di-heme active site. FEBS Lett..

[19] Sun Y-H (2012). The small protein CydX is required for function of cytochrome bd oxidase in Brucella abortus. Front. Cell. Infect. Microbiol.

[20] Borisov VB (2011). The cytochrome bd respiratory oxygen reductases. Biochim. Biophys. Acta (BBA)—Bioenerg..

[21] Belevich I (2007). Cytochrome bd from Azotobacter vinelandii: evidence for high-affinity oxygen binding. Biochemistry.

[22] Jünemann S, Wrigglesworth JM (1995). Cytochrome bd oxidase from Azotobacter vinelandii purification and quantitation of ligand binding to the oxygen reduction site. J. Biol. Chem..

[23] Arutyunyan AM (2012). Optical and magneto-optical activity of cytochrome bd from Geobacillus thermodenitrificans. Biochim. Biophys. Acta (BBA) - Bioenerg..

[24] Hao W (2006). Asymmetrical evolution of cytochrome bd subunits. J. Mol. Evol..

[25] Sharma V (2017). Insights into functions of the H channel of cytochrome c oxidase from atomistic molecular dynamics simulations. PNAS.

[26] Theßeling A (2020). The long Q‐loop of Escherichia coli cytochrome bd oxidase is required for assembly and structural integrity. FEBS Lett..

[27] Goojani HG (2020). The carboxy-terminal insert in the Q-loop is needed for functionality of Escherichia coli cytochrome bd-I. Biochim. Biophys. Acta (BBA)—Bioenerg..

[28] Sviriaeva E (2020). Features and functional importance of key residues of the mycobacterium tuberculosis cytochrome bd oxidase. ACS Infect. Dis..

[29] Bashiri G (2010). Metabolic engineering of cofactor F420 production in Mycobacterium smegmatis. PLOS One..

[30] Casadaban MJ (1980). Analysis of gene control signals by DNA fusion and cloning in Escherichia coli. J. Mol. Biol..

[31] Ritchie TK (2009). Reconstitution of membrane proteins in phospholipid bilayer nanodiscs. Methods Enzymol..

[32] Zheng SQ (2017). Anisotropic correction of beam-induced motion for improved single-particle electron cryo-microscopy. Nat. Methods.

[33] Rohou A, Grigorieff N (2015). CTFFIND4: fast and accurate defocus estimation from electron micrographs. J. Struct. Biol..

[34] Wagner T (2019). SPHIRE-crYOLO is a fast and accurate fully automated particle picker for cryo-EM. Commun. Biol..

[35] Scheres SHW (2012). RELION: implementation of a Bayesian approach to cryo-EM structure determination. J. Struct. Biol..

[36] Emsley P (2010). Features and development of Coot. Acta Crystallogr. Sect. D. Biol. Crystallogr..

[37] Adams PD (2010). PHENIX: a comprehensive Python-based system for macromolecular structure solution. Acta Crystallogr. Sect. D. Biol. Crystallogr..

[38] Chen VB (2010). MolProbity: all-atom structure validation for macromolecular crystallography. Acta Crystallogr. Sect. D. Biol. Crystallogr..

[39] Goddard TD (2018). UCSF ChimeraX: meeting modern challenges in visualization and analysis. Protein Sci..

[40] Sehnal D (2013). MOLE 2.0: advanced approach for analysis of biomacromolecular channels. J. Cheminform..

[41] Holm L, Rosenström P (2010). Dali server: conservation mapping in 3D.. Nucleic Acid Res..

[42] Wu EL (2014). CHARMM‐GUI Membrane Builder toward realistic biological membrane simulations. J. Comput. Chem..

[43] Olsson MHM (2011). PROPKA3: consistent treatment of internal and surface residues in empirical pKa predictions. J. Chem. Theory Comput..

[44] Best RB (2012). Optimization of the additive CHARMM all-atom protein force field targeting improved sampling of the backbone ϕ, ψ and side-chain χ1 and χ2 dihedral angles. J. Chem. Theory Comput..

[45] Kern NR (2014). Lipid-linked oligosaccharides in membranes sample conformations that facilitate binding to oligosaccharyltransferase. Biophys. J..

[46] Jorgensen WL (1998). Comparison of simple potential functions for simulating liquid water. J. Chem. Phys..

[47] Klauda JB (2010). Update of the CHARMM all-atom additive force field for lipids: validation on six lipid types. J. Phys. Chem. B..

[48] Mayne CG (2013). Rapid parameterization of small molecules using the force field toolkit. J. Comput. Chem..

[49] Visual VMD (1996). molecular dynamics. J. Mol. Graph..

[50] Agrawal NM, Denning EJ, Woolf TB, Beckstein O (2011). MDAnalysis: a toolkit for the analysis of molecular dynamics simulations. J. Comput. Chem..

[51] GROMACS: High performance molecular simulations through multi-level parallelism from laptops to supercomputers. (2015). SoftwareX.

[52] Darden T (1998). Particle mesh Ewald: an N⋅log(N) method for Ewald sums in large systems. J. Chem. Phys..

[53] Hess B (1997). LINCS: a linear constraint solver for molecular simulations. J. Comput. Chem..

[54] Berendsen HJC (1998). Molecular dynamics with coupling to an external bath. J. Chem. Phys..

[55] Hoover WG (1985). Canonical dynamics: Equilibrium phase-space distributions. Phys. Rev. A..

[56] Parrinello M, Rahman A (1998). Polymorphic transitions in single crystals: a new molecular dynamics method. J. Appl. Phys..

