# Supplementary material for: The cryo-EM structure of the bd oxidase from M. tuberculosis reveals a unique structural framework and enables rational drug design to combat TB
Source: Nat Commun. 2021 Sep 2;12:5236. doi: 10.1038/s41467-021-25537-z (PMC8413341; doi:10.1038/s41467-021-25537-z)
Supplement: Supplementary file 1 — Supplementary Information [file 41467_2021_25537_MOESM1_ESM.pdf]

The cryo-EM structure of the *bd* oxidase from *M. tuberculosis* reveals a unique structural framework and enables rational drug design to combat TB

Schara Safarian[1*], Helen K. Opel-Reading[2], Di Wu[1], Ahmad R. Mehdipour[3], Kiel Hards[4], Liam K. Harold[4], Melanie Radloff[1], Ian Stewart[5], Sonja Welsch[6], Gerhard Hummer[3,7], Gregory M. Cook[4], Kurt L. Krause[2], Hartmut Michel[1*]

Supplementary Table 1

Supplementary Figures 1 to 10

Supplementary References

Supplementary Table 1 - Cryo-EM data collection and validation. Cryo-EM data statistics of oxidized cytochrome *bd* oxidase from *M. tuberculosis*.

	as isolated	Aurachin D	AD3-11
**Data collection**			
Accession number	EMD-12451	EMD-12533	EMD-12532
Magnification	105k	105k	105k
Voltage / kV	300	300	300
Dose / e^{-}Å^{-2}	108	108	108
Pixel size / Å	0.837	0.837	0.837
Focus range / µm	-1.1. to -2.1	-1.1. to -2.1	-1.1. to -2.1
Recorded movies	12070	7401	8514
Final particle images	843799	194987	437753
Camera	Gatan K3	Gatan K3	Gatan K3
Microscope	Titan Krios G3i	Titan Krios G3i	Titan Krios G3i
**Image processing**			
Initial model	*de novo*	EMD-12451	EMD-12451
Resolution ($FSC_{0.143}$) / Å	2.5	3.3	2.8
Applied B-factor / Å^2	-70	-100	-80
**Model refinement**			
PDB accession	7NKZ		
Validation			
$FSC^{map\text{-}to\text{-}model}{}_{(0.5)}$ / Å	2.6		
MolProbity score	1.58		
Composition			
Atoms	6424		
Protein residues	792		
Waters	42		
Ligands	2 HEB, 1 HDD, 1 MK-9, 1 OXY		
Bonds (R.M.S.D.)			
Length (Å)	0.004		
Angles (°)	1.07		
B-factors (min/max/mean)			
Protein	29.4/88.68/50.67		
Ligand	33.33/45.54/39.28		
Waters	33.22/70.97/45.11		
Clashscore	11.51		
Ramachandran plot (%)			
Favored	98.85		
Allowed	1.15		
Outliers	0		
Rotamer outliers (%)	0.63		

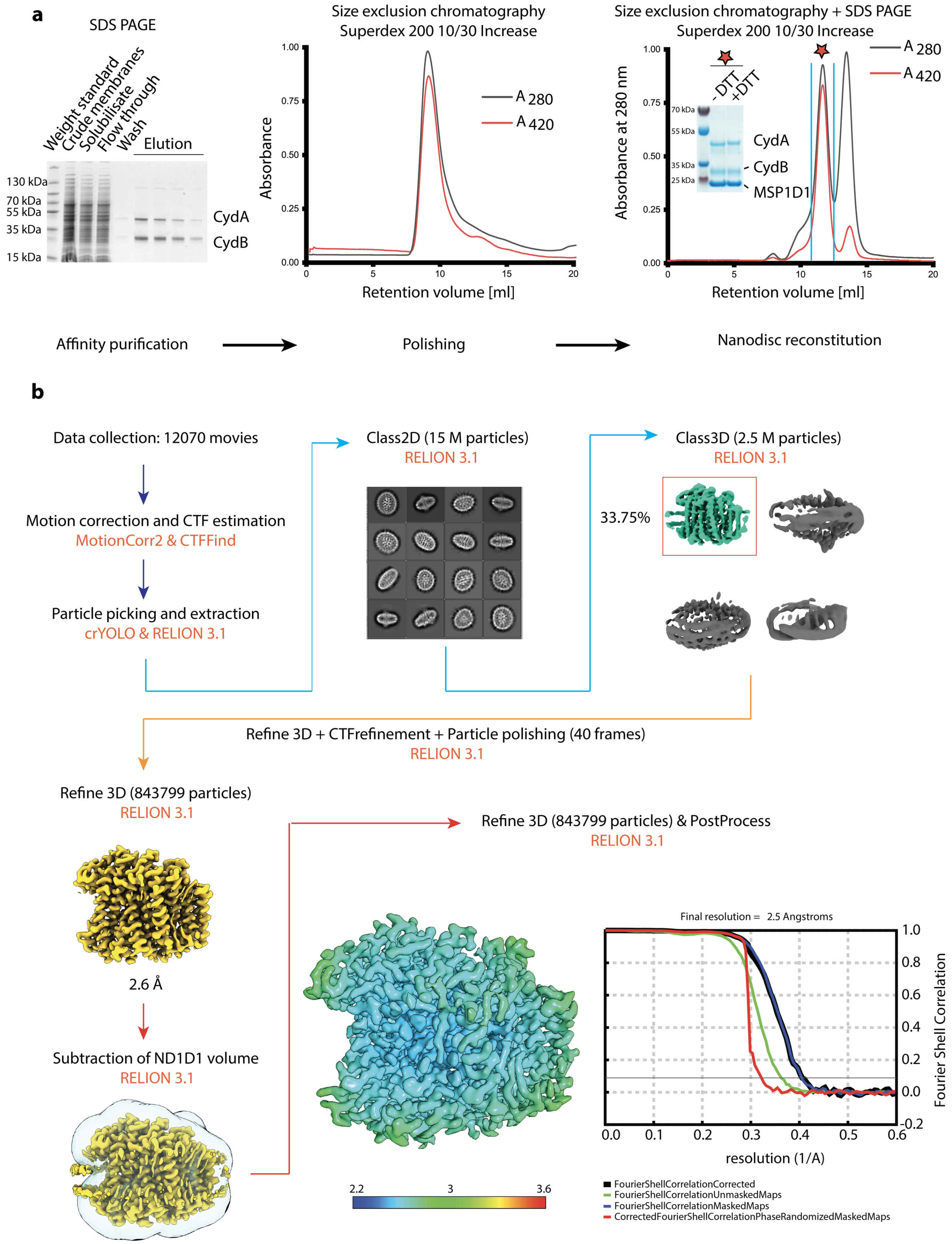
a
SDS PAGE
Weight standard
Crude membranes
Solubilisate
Flow through
Wash
Elution
130 kDa
70 kDa
55 kDa
35 kDa
15 kDa
CydA
CydB
Size exclusion chromatography
Superdex 200 10/30 Increase
Absorbance
A 280
A 420
Retention volume [ml]
Size exclusion chromatography + SDS PAGE
Superdex 200 10/30 Increase
Absorbance at 280 nm
- DTT
+DTT
70 kDa
55 kDa
35 kDa
25 kDa
CydA
CydB
MSP1D1
A 280
A 420
Retention volume [ml]
Affinity purification
Polishing
Nanodisc reconstitution
b
Data collection: 12070 movies
Motion correction and CTF estimation
MotionCorr2 & CTFFind
Particle picking and extraction
crYOLO & RELION 3.1
Class2D (15 M particles)
RELION 3.1
Class3D (2.5 M particles)
RELION 3.1
33.75%
Refine 3D + CTFrefinement + Particle polishing (40 frames)
RELION 3.1
Refine 3D (843799 particles)
RELION 3.1
2.6 Å
Subtraction of ND1D1 volume
RELION 3.1
Refine 3D (843799 particles) & PostProcess
RELION 3.1
2.2
3
3.6
Final resolution = 2.5 Angstroms
Fourier Shell Correlation
resolution (1/A)
FourierShellCorrelationCorrected
FourierShellCorrelationUnmaskedMaps
FourierShellCorrelationMaskedMaps
CorrectedFourierShellCorrelationPhaseRandomizedMaskedMaps

c

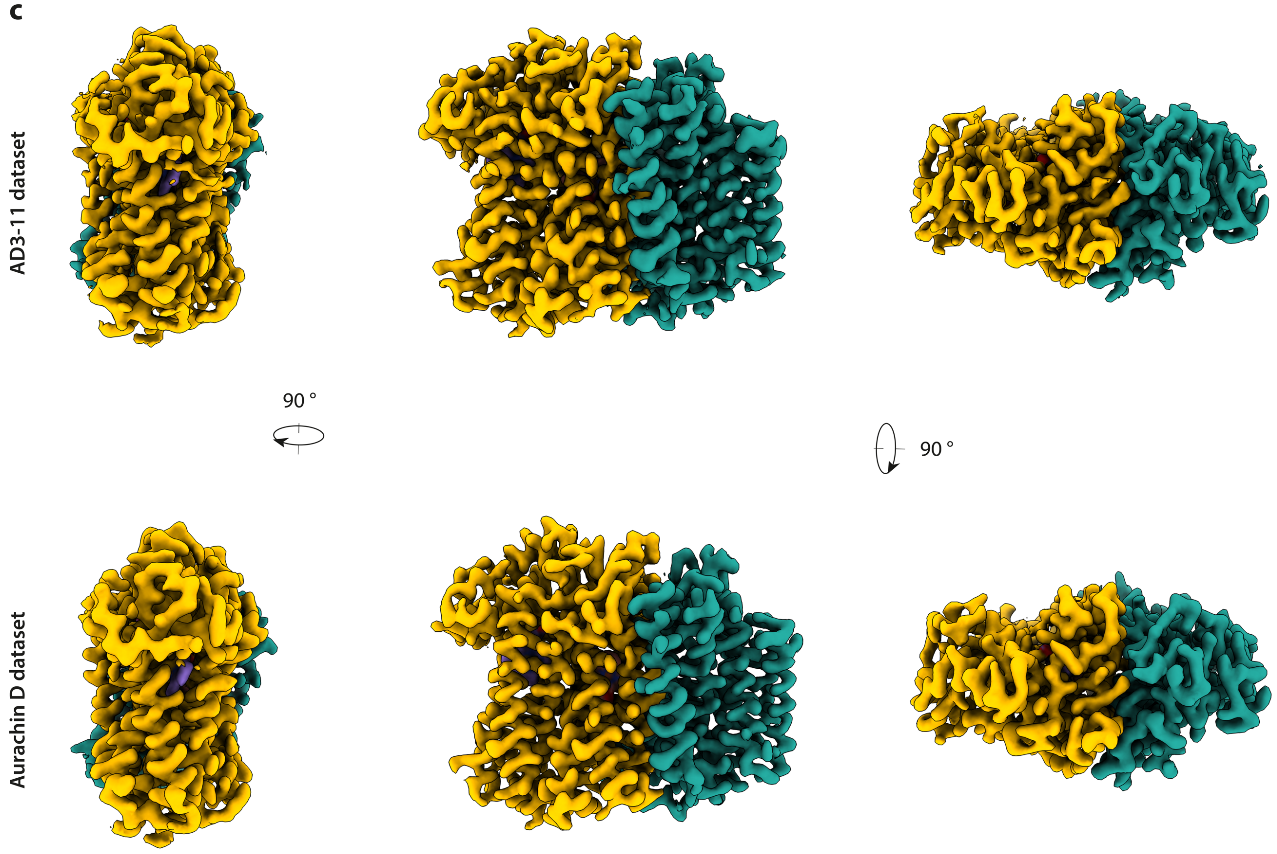

d

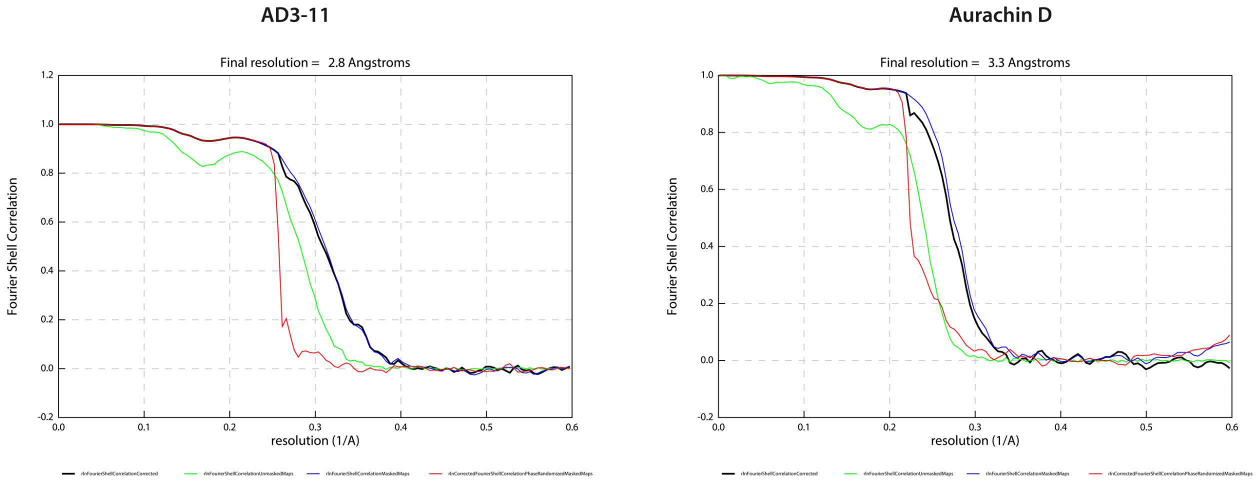

Supplementary Fig. 1 - Sample preparation and image processing. (**a**) The cytochrome *bd* oxidase was produced in *Mycobacterium smegmatis* mc^{2}155 Δ*cydAB* cells and purified by FLAG-tag affinity chromatography followed by SEC. The purified complex was reconstituted into lipid nanodiscs (1D1) and separated from empty nanodiscs by SEC. Peak fractions were collected and analyzed by SDS-PAGE (oxidized and reduced form) and used for downstream cryo-EM specimen preparation (**b**) All datasets (*as isolated*, Aurachin D, AD3-11) were processed according to the following scheme, which is shown for the *as isolated* dataset as an example. Statistical values for all three datasets are summarized in Supplementary Table 1. Initial full-frame motion correction was performed with MotionCorr2 (RELION-

3.1). CTF estimation was performed using CTFFind4 (version 4.1). A number of 15M Particles were picked using crYOLO and subsequently extracted in the RELION-3.1 suite. A total of 2.5 M particle images from 2D classes indicating distinct features were selected for further processing. A subset of these particles was used for initial model generation. Three-dimensional classification showed a single class exhibiting map features of a transmembrane protein. Particle images from class 1 were used for a consensus 3D refinement to an average resolution of $FSC_{0.143}$ = 2.9 Å. To further improve the resolution, CTF refinement, particle polishing, and nanodisc subtraction were performed. A final refinement step using fine angular sampling steps and a tight soft-edged 3D mask converged to a resolution of $FSC_{0.143}$ = 2.5 Å. The final reconstruction indicates differences in local resolution, with higher resolution in the center and slightly lower resolution at the periphery of the complex. (**c**) Unsharpened cryo-EM maps of *bd* oxidase structures in presence of inhibitors Aurachin D and AD3-11. (**d**) Fourier shell correlation plots of inhibitor datasets.

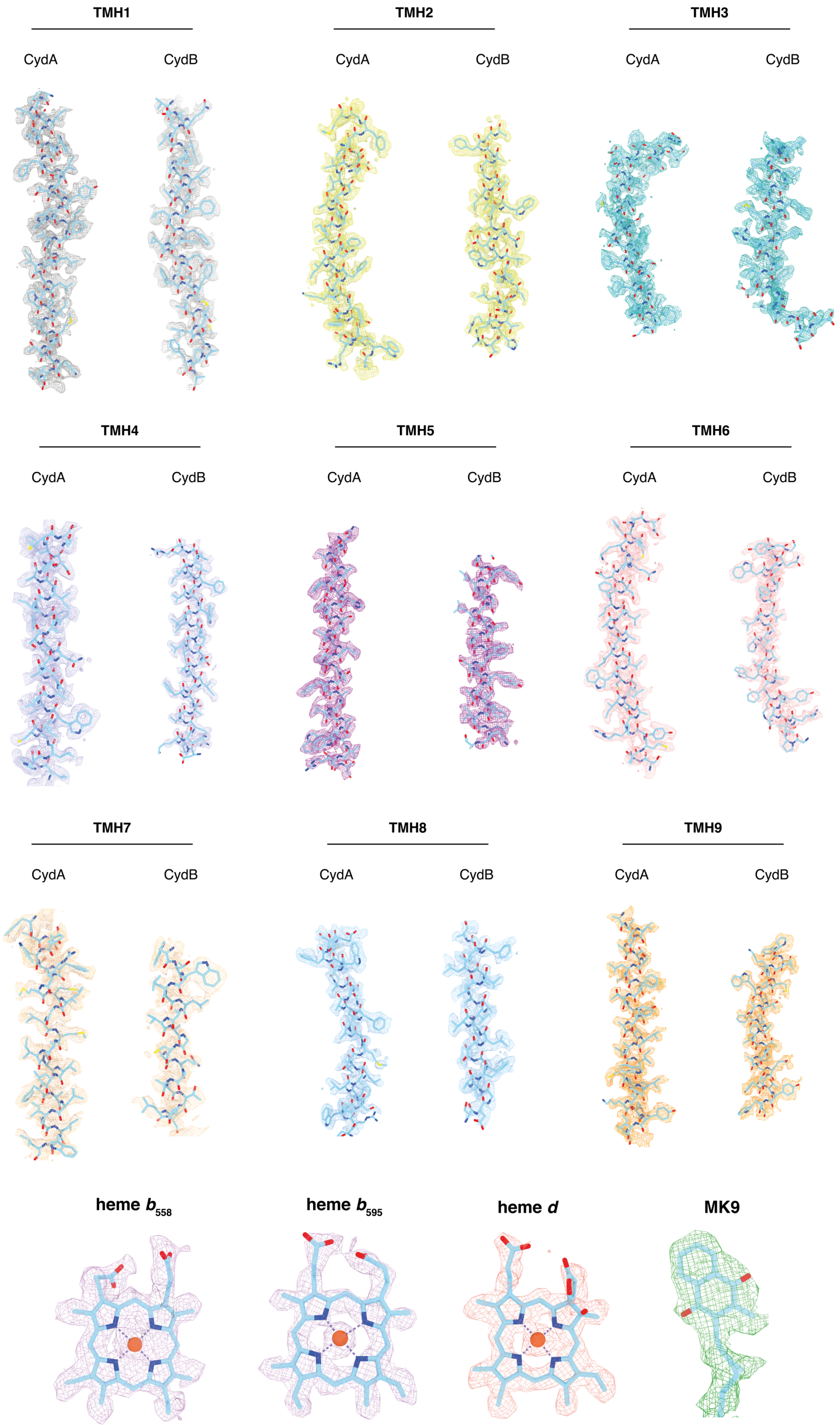

Supplementary Fig. 2 – Density features at 2.5 Å resolution. Visual inspection of density features of transmembrane helices and cofactors (heme b_{558}, heme b_{595}, heme *d,* and MK-9). Symmetry related transmembrane helices are presented in a matching color code. Helix topologies are maintained according to the *bd* oxidase fold. Presented densities are sharpened by a b-factor of -70.

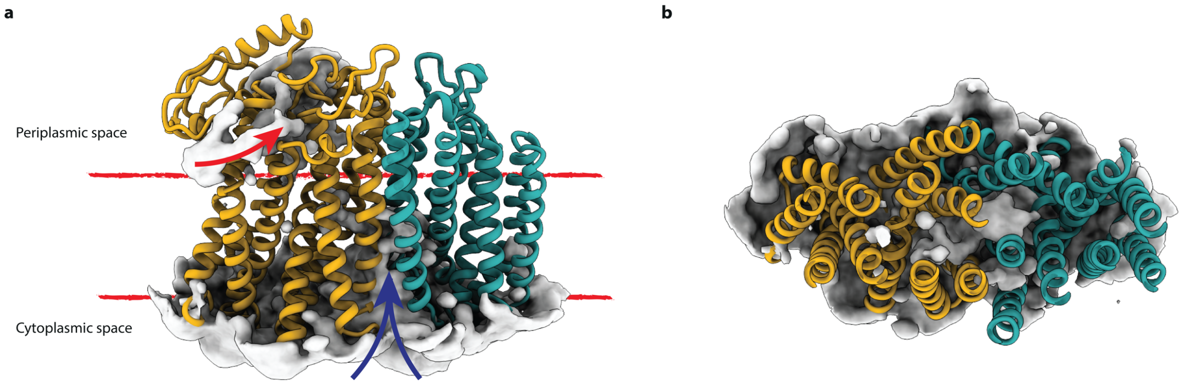

Supplementary Fig. 3 – Averaged protein hydration map obtained from molecular dynamics simulations. (**a**) Solvent accessibility from the cytoplasmic and periplasmic space. Entry sites of the substrate proton channel and the solvent accessible region near heme b_{558} are indicated by red and blue arrows, respectively. (**b**)Periplasmic view on the mycobacterial *bd* oxidase showing the pathway of solvent molecules towards the enzyme reaction center at the interface between CydA and CydB. CydA, yellow; CydB, green; solvent map, grey.

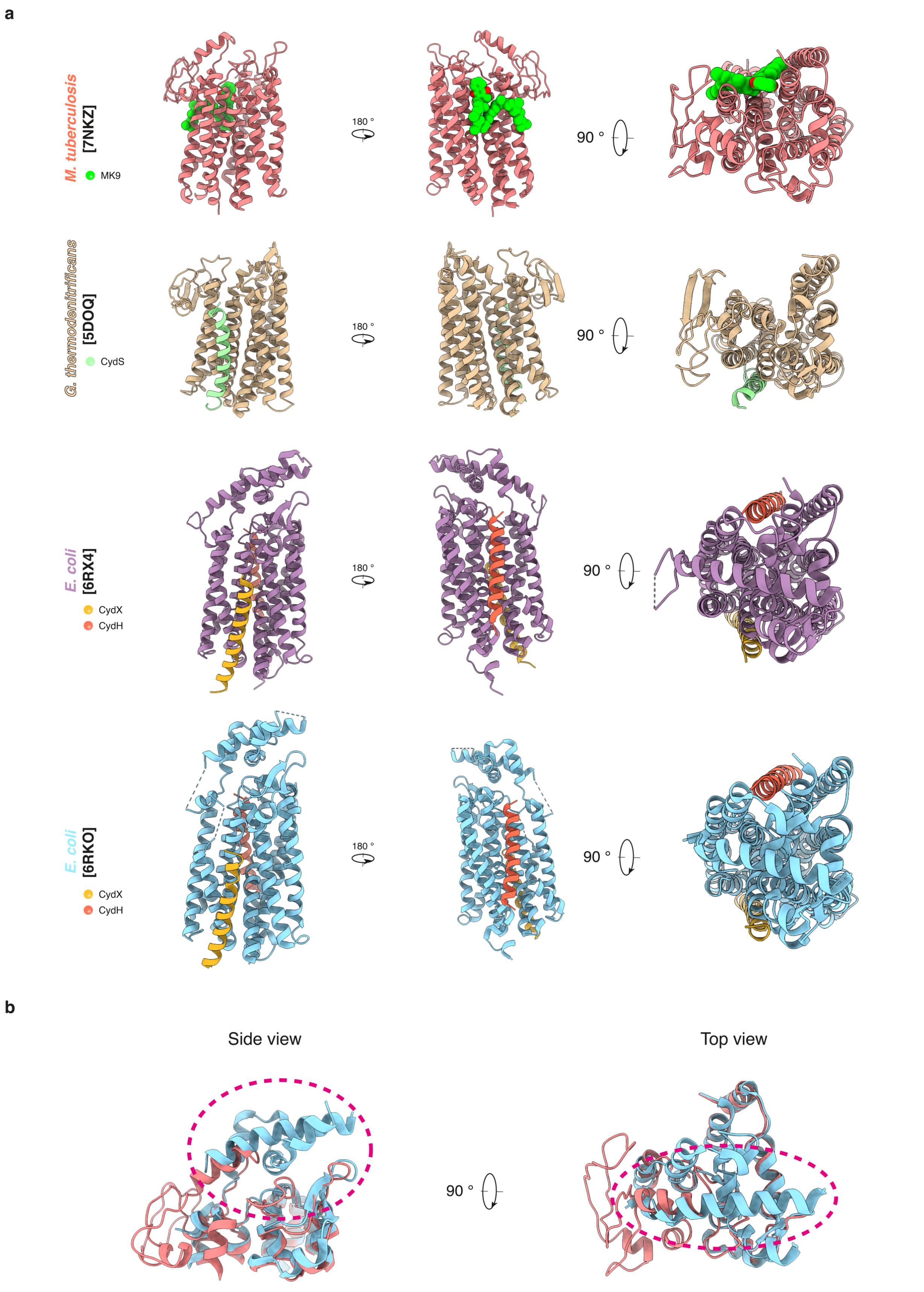
a
M. tuberculosis
[7NKZ]
MK9
180 °
90 °
G. thermodenitrificans
[5DOQ]
CydS
180 °
90 °
E. coli
[6RX4]
CydX
CydH
180 °
90 °
E. coli
[6RKO]
CydX
CydH
180 °
90 °
b
Side view
Top view
90 °

c

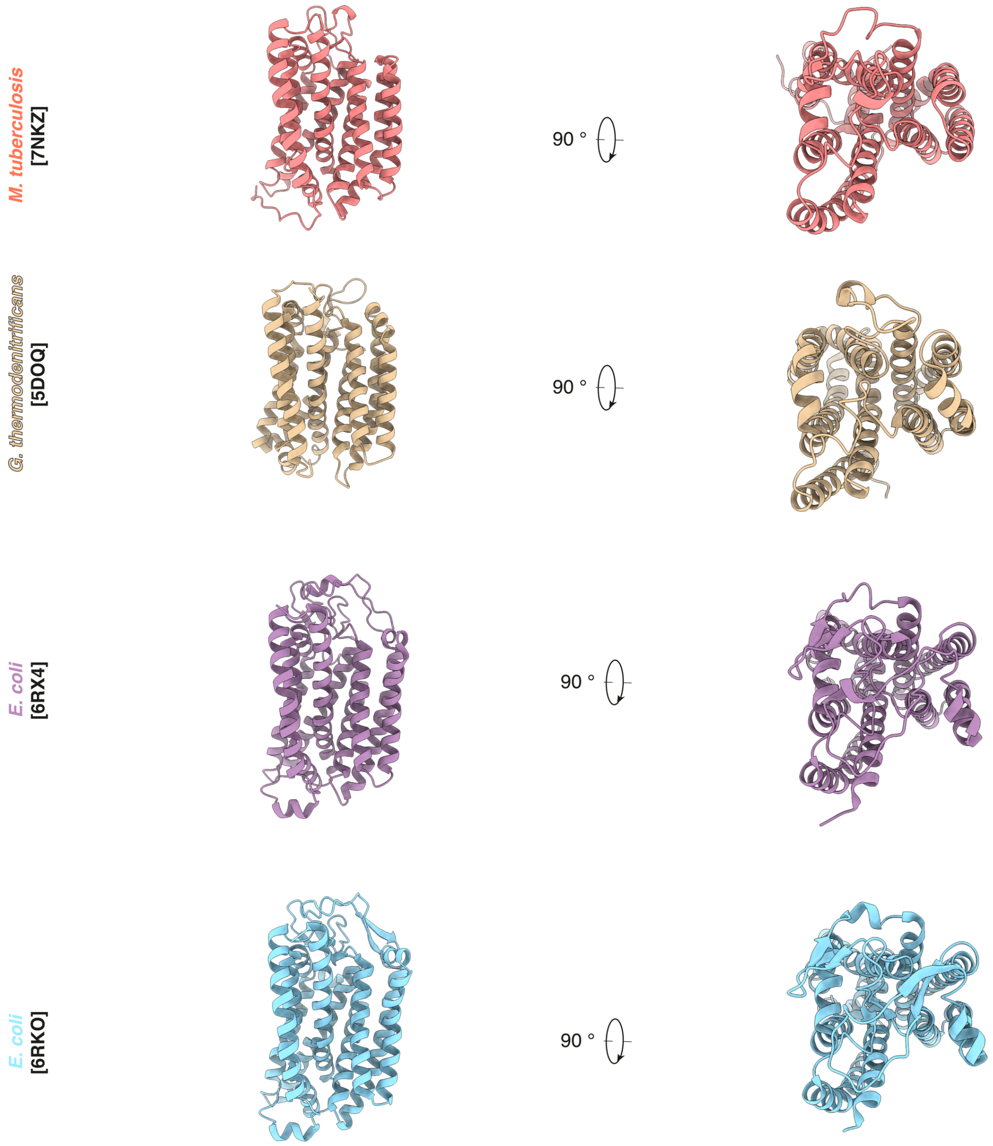

d

	6RKO – CydA (*E. coli*)		6RX4 – CydA (*E. coli*)		5DOQ – CydA (*G. th*)	
	R.M.S.D	Z-score	R.M.S.D	Z-score	R.M.S.D	Z-score
CydA (*M.tb*)	1.6	45.2	1.7	47.4	2.4	42.1
	6RKO – CydB (*E. coli*)		6RX4 – CydB (*E. coli*)		5DOQ – CydB (*G. th*)	
CydB (*M.tb*)	1.8	41.8	1.8	42	2.7	28.8

Supplementary Fig. 4 – Fold analysis and structural alignments

(**a, c**) Folds of CydA and CydB subunits from proteobacterial, actinobacterial and firmicute cytochrome *bd* oxidase structures shown as ribbon models. Unique structural components such as accessory single-transmembrane helix subunits or bound quinones are shown: MK9 = green, CydS = pale green, CydX = yellow, CydH = red. (**b**) Structural alignment of Q-loop domains from *E. coli* and *M. tuberculosis*. The highlighted region (dotted purple circles) indicates the location and topology of the respective Q_c-loops. (**d**) Root mean square deviations (R.M.S.D) of Cα positions and corresponding Z-scores obtained from alignments of *bd* oxidase structures.

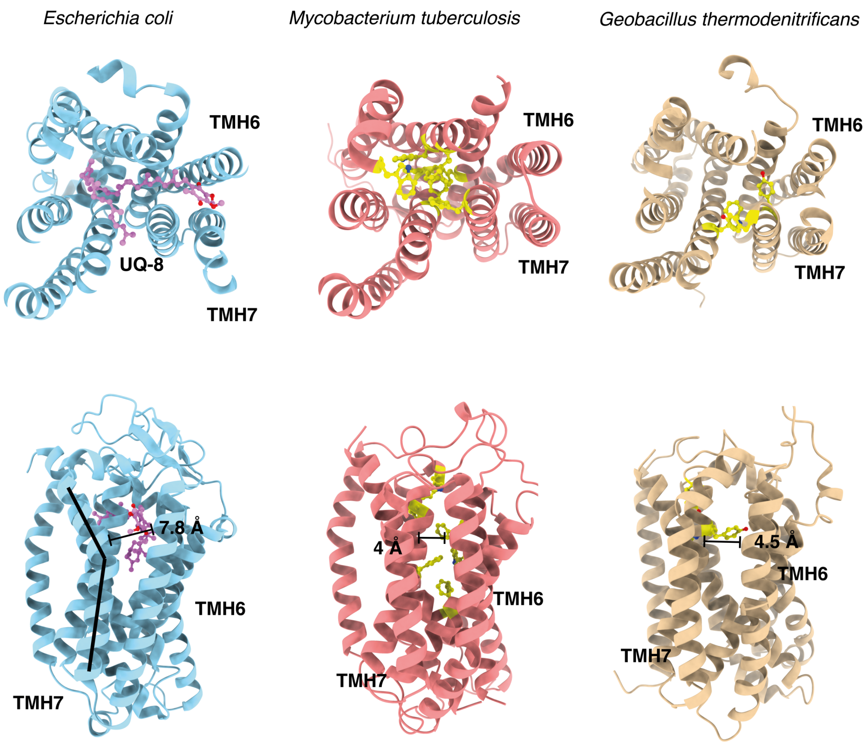

Supplementary Fig. 5 – Architecture of CydB subunits. Ribbon representation of CydB subunits from *E. coli* (blue), *M. tuberculosis* (red), and *G. thermodenitrificans* (beige) in top view (upper panel) and side view (lower panel) orientations. Aromatic residues positioned within the CydB specific large membrane cavity are shown as yellow stick-and-ball models. The *E. coli* specific UQ-8 molecule that occupies the membrane cavity of CydB is shown as a purple ball-and-stick model. Distances between TMH6 and TMH7 are indicated. The large distances between these two helices is bridged by the UQ-8 head group in the *E. coli* enzyme. This inter-helix distance is significantly shorter in the *bd* oxidase structures of *M. tuberculosis* and *G. thermodenitrificans*. The mycobacterial enzyme shows a larger number of stabilizing van-der-Waals interactions within the membrane cavity than the enzyme from *G. thermodenitrificans*.

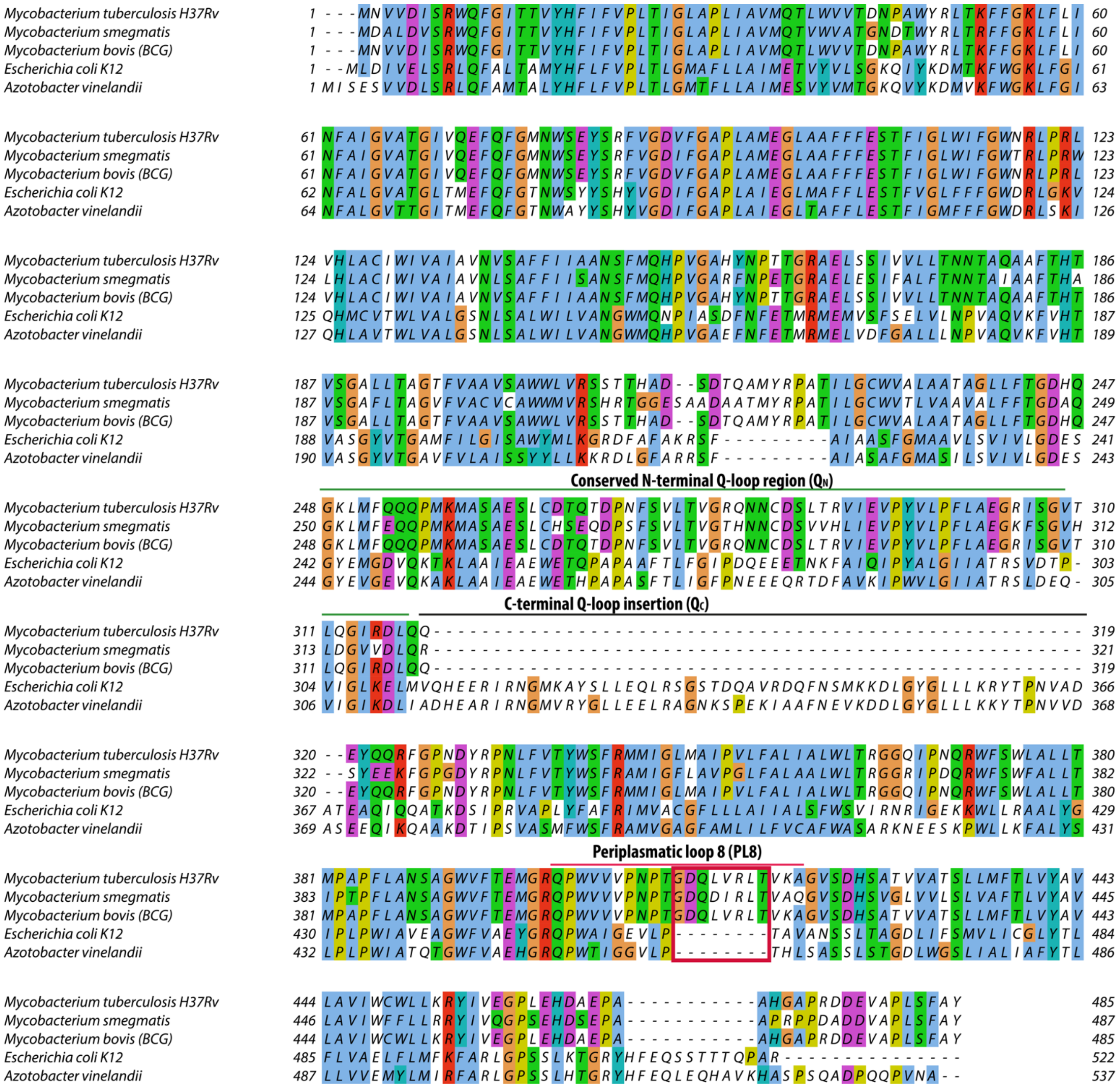

Supplementary Fig. 6 – Sequence alignment of selected CydA subunits. Sequence alignments of selected CydA homologs from mycobacterial cytochrome *bd* oxidase and proteobacterial enzymes containing a long Q-loop variant. Q_N and Q_C regions are indicated by green and black bars, respectively. The periplasmic loop 8 (PL8) which shows an interaction with the Q_C domain of cyt. $bd_{M.tb}$ is indicated by a red bar. The mycobacterium specific C-terminal PL8 insertion is highlighted by a red box. Sequence alignments were generated using Clustal Omega[1].

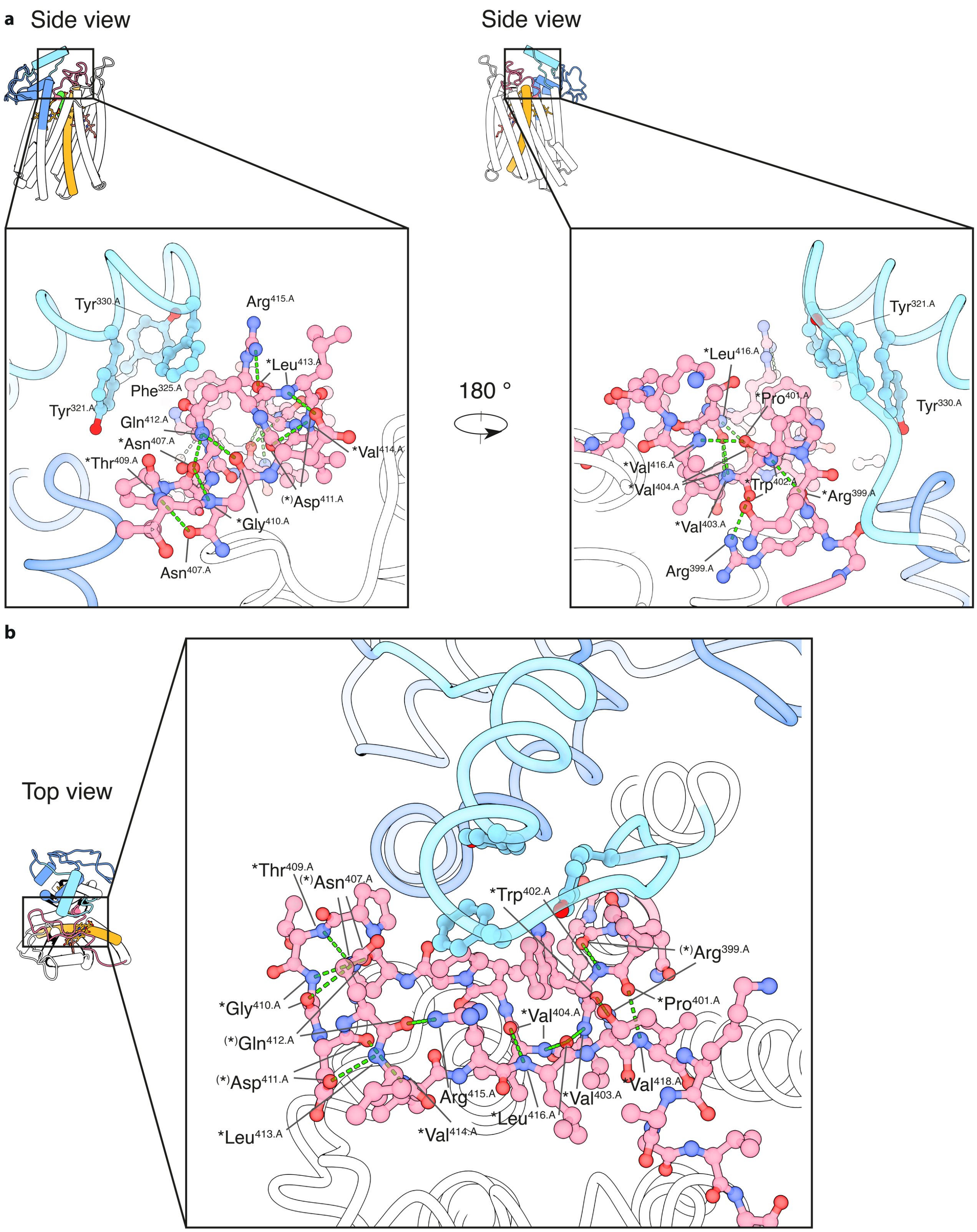

Supplementary Fig. 7 – Stabilizing hydrogen bond network of PL8. (**a**) Side view and (**b**) top view orientations of periplasmic loop 8 of CydA. Residues of PL8 are presented as pink ball-and-stick models. Hydrogen bonds are indicated with dashed green lines. Amino acid residue participating in the PL8 hydrogen bond network are indicated. Asterisks refer to backbone oxygens and amides as H-bond donors and acceptors. Brackets indicate participation of backbone and sidechain atoms in H-bond formation.

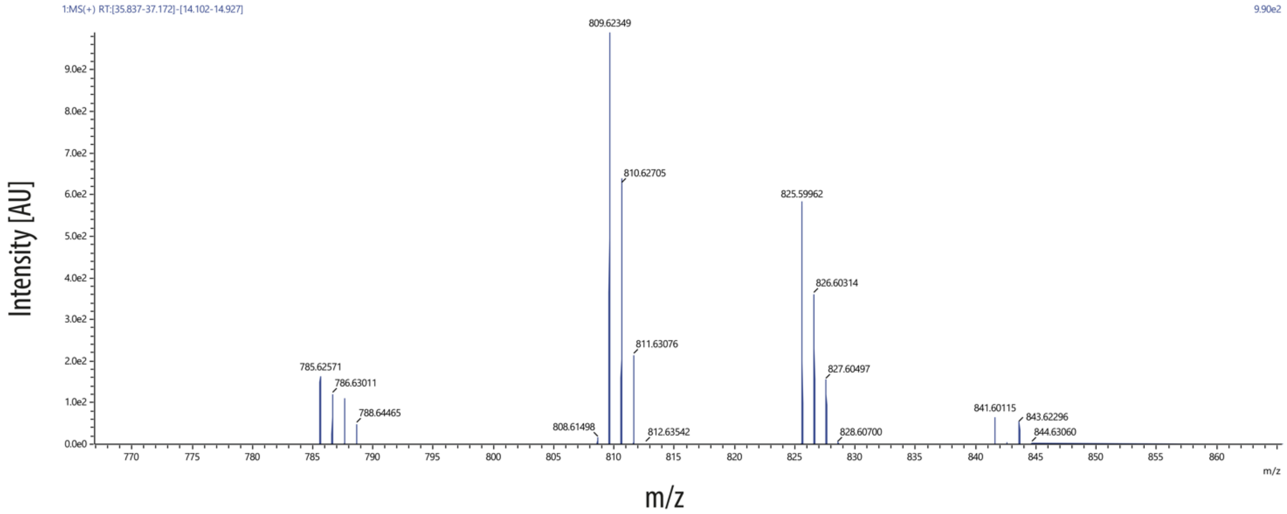

Supplementary Fig. 8 – LC-MS spectrum of identified MK9 species in the cytochrome *bd* oxidase. Peaks at m/z 785.6, 809.6, and 825.6 display H^+, Na^+ and K^+ adducts of oxidized MK9, respectively.

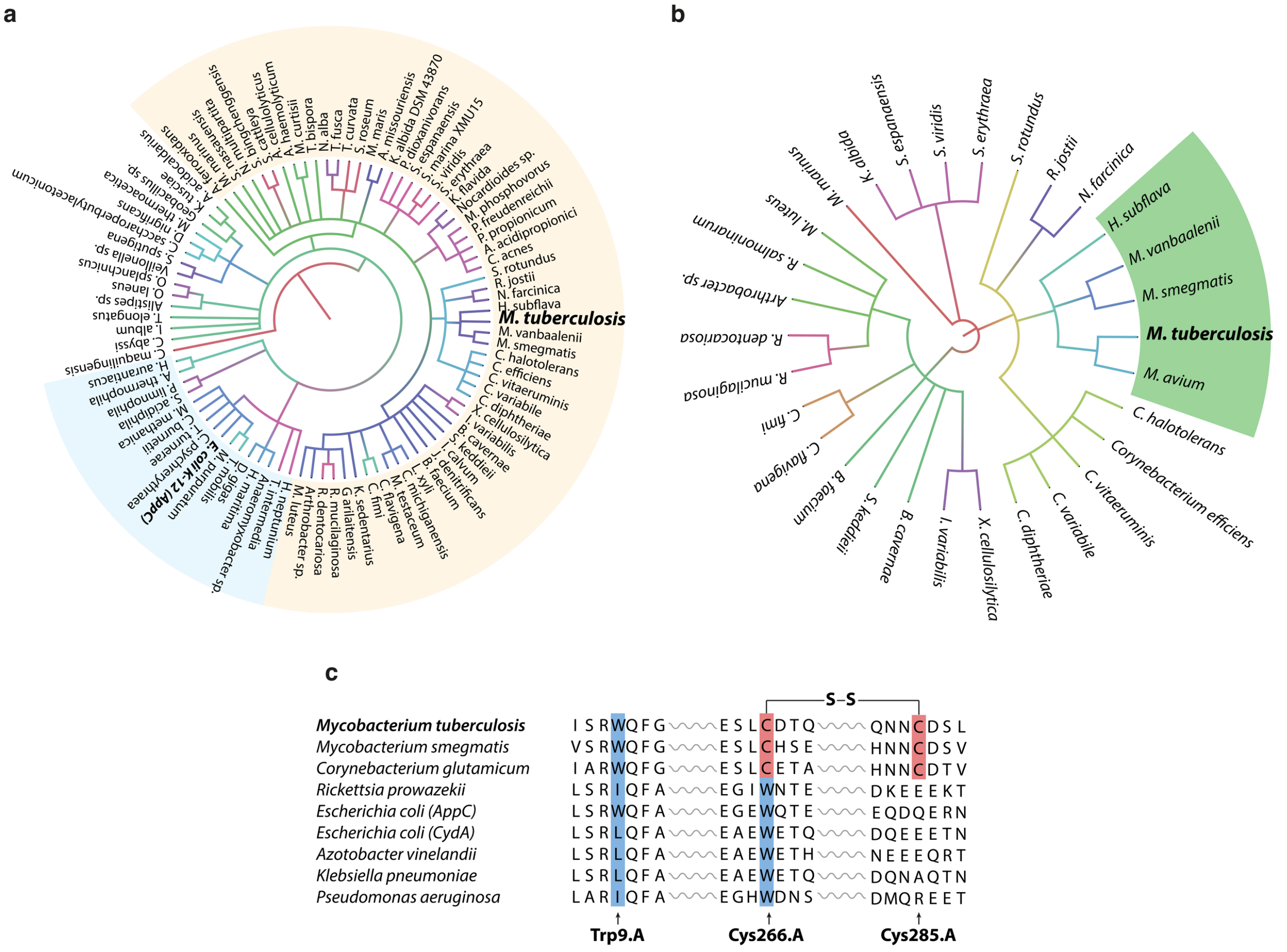

Supplementary Fig. 9 – Phylogenetic distribution of structural features involved in allosteric and thiol modulation of cytochrome *bd* oxidase activity.

Multiple sequence alignments were carried out using 561 CydA sequences from the manually curated representative genome list (Seed) of the protein family database (Pfam). (**a**) Phylogenetic distribution of Trp$^{9.A}$ residue among bacterial and archaeal representative genomes. The majority of these sequences belong to the phylum of Actinobacteria (beige). Conservation of the Trp residue was also found in Proteobacteria although with lesser representation (blue). (**b**) Phylogenetic distribution of the disulfide forming Cys$^{266.A}$ – Cys$^{285.A}$ pair. Among the total number of analyzed sequences only 30 orthologs (5.7 %) exhibited this residue pair. Intriguingly, all of these sequences belong the phylum of Actinobacteria, including mycobacteria (green), and also show the presence of the signature Trp residue in TMH1. (**c**) Multiple sequence alignment of selected cytochrome *bd* oxidases. Positions of Trp$^{9.A}$, Cys$^{266.A}$, and Cys$^{285.A}$ residues are indicated. Disulfide bond forming cysteines are highlighted in red.

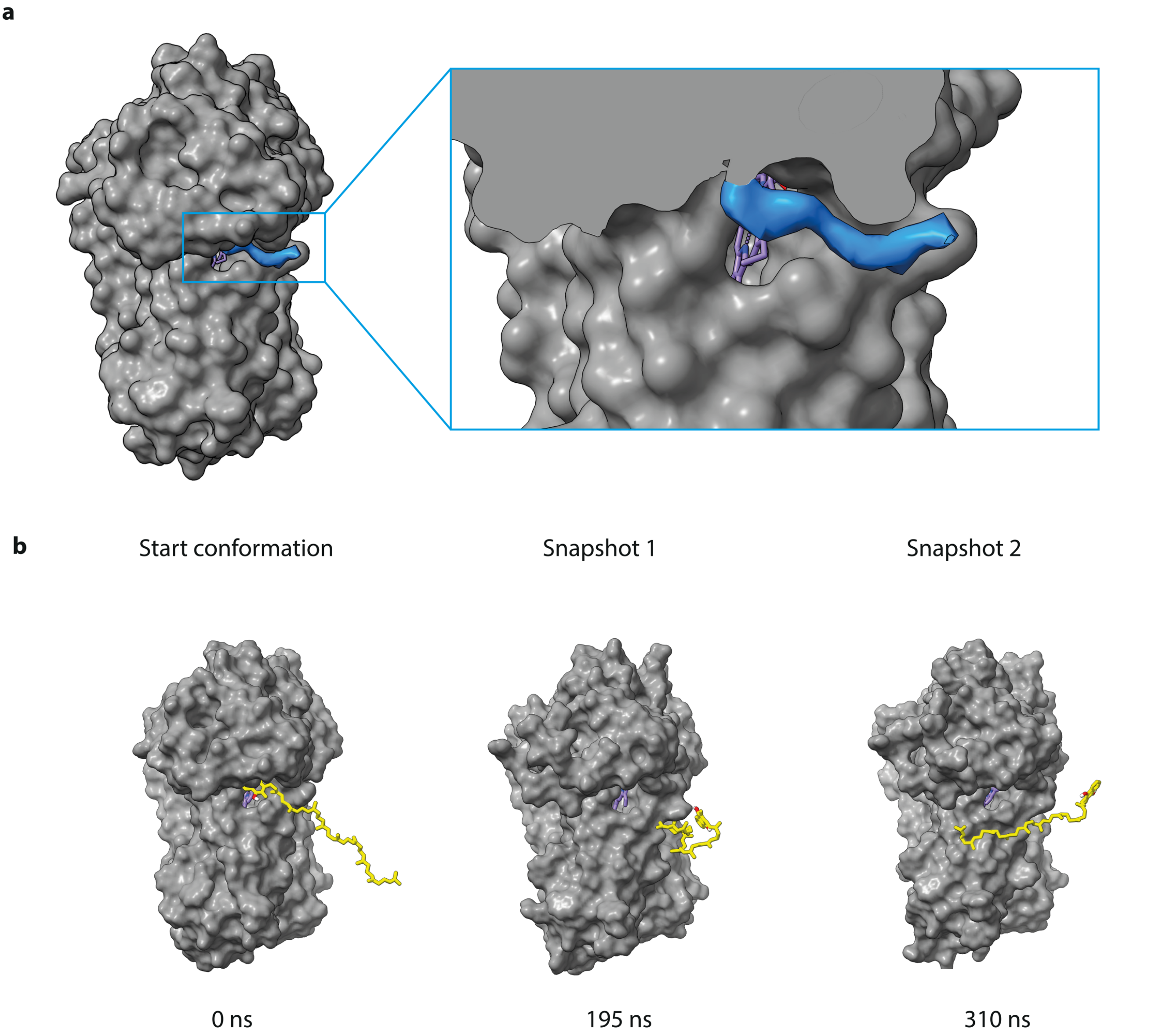

Supplementary Fig. 10 – MD simulation of reduced MK-9 binding to the Q-loop domain. (a) Representative snapshots of a 750 ns MD simulation with reduced MK-9 docked closely to the interface between the Q_N region and heme b_{595}. The head group was placed within an observable density feature at this location. **(b)** The simulation snapshots visualized here demonstrate that reduced MK-9 does not form a stable interaction in the region around the b-type heme with the Q-loop domain. The quinol molecule diffuses into the lipid bilayer during the time frame of our simulation. CydA is shown as a grey surface mode Reduced MK-9 is shown as a yellow stick model. Heme b_{558} is shown as a magenta stick model.

Supplementary References

1. F. Sievers *et al.*, Fast, scalable generation of high-quality protein multiple sequence alignments using Clustal Omega. *Molecular Systems Biology*. **7**, 539–539 (2011).
