# Supplementary material for: The cryo-EM structure of the bd oxidase from M. tuberculosis reveals a unique structural framework and enables rational drug design to combat TB
Source: Nat Commun. 2021 Sep 2;12:5236. doi: 10.1038/s41467-021-25537-z (PMC8413341; doi:10.1038/s41467-021-25537-z)
Supplement: Supplementary file 2 — Reporting summary [file 41467_2021_25537_MOESM2_ESM.pdf]

nature research

Corresponding author(s): Dr. Schara Safarian
Prof. Dr. Dr. Hartmut Michel

Last updated by author(s): Aug 10, 2021

Reporting Summary

Nature Research wishes to improve the reproducibility of the work that we publish. This form provides structure for consistency and transparency in reporting. For further information on Nature Research policies, see our Editorial Policies and the Editorial Policy Checklist.

Statistics

For all statistical analyses, confirm that the following items are present in the figure legend, table legend, main text, or Methods section.

n/a	Confirmed	
☐	☒	The exact sample size (*n*) for each experimental group/condition, given as a discrete number and unit of measurement
☐	☒	A statement on whether measurements were taken from distinct samples or whether the same sample was measured repeatedly
☒	☐	The statistical test(s) used AND whether they are one- or two-sided<br>*Only common tests should be described solely by name; describe more complex techniques in the Methods section.*
☒	☐	A description of all covariates tested
☒	☐	A description of any assumptions or corrections, such as tests of normality and adjustment for multiple comparisons
☐	☒	A full description of the statistical parameters including central tendency (e.g. means) or other basic estimates (e.g. regression coefficient) AND variation (e.g. standard deviation) or associated estimates of uncertainty (e.g. confidence intervals)
☒	☐	For null hypothesis testing, the test statistic (e.g. *F*, *t*, *r*) with confidence intervals, effect sizes, degrees of freedom and *P* value noted<br>*Give P values as exact values whenever suitable.*
☐	☒	For Bayesian analysis, information on the choice of priors and Markov chain Monte Carlo settings
☒	☐	For hierarchical and complex designs, identification of the appropriate level for tests and full reporting of outcomes
☒	☐	Estimates of effect sizes (e.g. Cohen's *d*, Pearson's *r*), indicating how they were calculated

Our web collection on statistics for biologists contains articles on many of the points above.

Software and code

Policy information about availability of computer code

Data collection	Titan Krios microscope operated at 300 kV and equipped with a BioQuantum energy filter and a K2 camera (Gatan); Data collection quality was monitored through EPU v. 2.9 and CryoSparc Live v. 3.0.1.
Data analysis	ChimeraX v.1.1, MotionCor2, CTFFind-4.1, CLUSTAL Omega v.1.2.4 , RELION-3.1; CryoSPARC Live v.3.0.1; COOT v. 0.8.9; Phenix v. 1.14; MolProbity v. 4.5; CHARMM36m force field was used for protein, lipids, and ions, together with TIP3P water; Visual Molecular Dynamics (VMD) v. 1.9.2; GROMACS v. 2019.6, EPU v. 2.9; FigTree v.1.4.4.

For manuscripts utilizing custom algorithms or software that are central to the research but not yet described in published literature, software must be made available to editors and reviewers. We strongly encourage code deposition in a community repository (e.g. GitHub). See the Nature Research guidelines for submitting code & software for further information.

Data

Policy information about availability of data

All manuscripts must include a data availability statement. This statement should provide the following information, where applicable:

- Accession codes, unique identifiers, or web links for publicly available datasets
- A list of figures that have associated raw data
- A description of any restrictions on data availability

The Cryo-EM maps of the cytochrome bd oxidase from M. tuberculosis are deposited at the Electron Microscopy Data Bank (https://www.ebi.ac.uk/emdb) under accession numbers EMD-12451, EMD-12532, EMD-12533. The model of the cytochrome bd oxidase structure was submitted to the Protein Data Base (https://www.wwpdb.org/) with accession number 7NKZ. The DALI database (http://ekhidna2.biocenter.helsinki.fi/dali/) was used to perform structural alignments of PDB entries with the accession numbers 5DOQ, 6RKO, 6Rx4, 7NKZ. The protein family database (PFAM) was used to retrieve manually curated representative CydA sequences from 561 bacterial species (https://pfam.xfam.org/family/Cyt_bd_oxida_I).

Field-specific reporting

Please select the one below that is the best fit for your research. If you are not sure, read the appropriate sections before making your selection.

☒ Life sciences ☐ Behavioural & social sciences ☐ Ecological, evolutionary & environmental sciences

For a reference copy of the document with all sections, see nature.com/documents/nr-reporting-summary-flat.pdf

Life sciences study design

All studies must disclose on these points even when the disclosure is negative.

Sample size	Sample sizes (number of collected micrographs) of respective cryo-EM datasets were chosen based on instrument availability and applied dose rate. Datasets of > 5000 micrographs for a respective sample ensured a sufficient number of particles to achieve resolutions < 3 Å. The smallest collected dataset contained 7401 micrographs (Aurachin D sample) while the largest dataset contained 12070 micrographs (as isolated sample). Oxygen consumption measurements were performed in technical replicates (n = 7 for IMV containing overproduced cytochrome bd oxidase, n=3 for control IMVs from ΔcydAB cells containing an empty expression vector). Technical replicates were chosen to determine standard deviation values for each data points and to validate data quality.
Data exclusions	No data were excluded.
Replication	Simple particle cryo-EM is based on averaging protein particles of nearly identical orientation within a vitreous layer of ice. Therefore, replication is not per se required to ensure statistical robustness of structural data. In case of this work, we have determined three individual structures of cytochrome bd under different sample conditions. In all of these structures, the general architecture of cytochrome bd remained identical. Hence these data can be considered as biological replicates of the presented structural data.
Randomization	Generally, no randomization was required for the experimental design of this study. However, it is to note that particles are randomized during data processing steps in Relion-3.1 (randomization during 2D classification, randomized half sets during Refine3D). Randomized half sets of particles are used in final reconstruction steps in order to determined gold-standard Fourier shell correlations based on the 0.143 level.
Blinding	This study was performed on a single enzyme sample. The experimental design did not require for blinding approaches in order to ensure robustness of obtained structural and biochemical data.

Reporting for specific materials, systems and methods

We require information from authors about some types of materials, experimental systems and methods used in many studies. Here, indicate whether each material, system or method listed is relevant to your study. If you are not sure if a list item applies to your research, read the appropriate section before selecting a response.

Materials & experimental systems

n/a	Involved in the study	
☒	☐	Antibodies
☒	☐	Eukaryotic cell lines
☒	☐	Palaeontology and archaeology
☒	☐	Animals and other organisms
☒	☐	Human research participants
☒	☐	Clinical data
☒	☐	Dual use research of concern

Methods

n/a	Involved in the study	
☒	☐	ChIP-seq
☒	☐	Flow cytometry
☒	☐	MRI-based neuroimaging
